# Ethylene and Metal Stress: Small Molecule, Big Impact

**DOI:** 10.3389/fpls.2016.00023

**Published:** 2016-02-02

**Authors:** Els Keunen, Kerim Schellingen, Jaco Vangronsveld, Ann Cuypers

**Affiliations:** Environmental Biology, Centre for Environmental Sciences, Hasselt UniversityDiepenbeek, Belgium

**Keywords:** ethylene, metals, oxidative stress, signal transduction, crosstalk

## Abstract

The phytohormone ethylene is known to mediate a diverse array of signaling processes during abiotic stress in plants. Whereas many reports have demonstrated enhanced ethylene production in metal-exposed plants, the underlying molecular mechanisms are only recently investigated. Increasing evidence supports a role for ethylene in the regulation of plant metal stress responses. Moreover, crosstalk appears to exist between ethylene and the cellular redox balance, nutrients and other phytohormones. This review highlights our current understanding of the key role ethylene plays during responses to metal exposure. Moreover, particular attention is paid to the integration of ethylene within the broad network of plant responses to metal stress.

## Setting the scene

With the global population exceeding nine billion by 2050, it is of increasing importance to optimize plant growth and ensure food and feed supply. However, plant yield is severely affected by environmental stress factors such as drought, nutrient deficiency, salinity and metal pollution (Mittler, 2006; Dolferus, 2014). Toxic metals and metalloids accumulate in the environment because of industrial applications. Contamination peaks occurred throughout history (e.g. the Roman Empire and Industrial Revolution) and current production rates are still high. In addition, the contribution of metal-contaminated fertilizers, pesticides and sewage sludge to overall metal pollution should not be ignored (Alloway, 2012). Metals such as cadmium (Cd), mercury (Hg) or lead (Pb) are not essential for plants. Therefore, even low concentrations interfere with plant growth and development and cause significant yield losses worldwide. On the other hand, excess levels of essential micronutrients such as copper (Cu), iron (Fe), nickel (Ni) and zinc (Zn) are phytotoxic as well (Cuypers et al., 2009; Hänsch and Mendel, 2009).

Plants are primary producers and therefore constitute an important bridge between the soil elemental composition and the food chain. Non-essential trace elements such as As and Cd opportunistically enter plant tissues via the same transport systems used to take up essential nutrients (Verbruggen et al., 2009; Seth et al., 2012). Excessive accumulation of toxic metals in food and feed crops represents a severe threat to human health (Järup, 2003), indicating the need to remediate metal-contaminated soils. However, recent efforts regarding the use of plants to clean-up soils via phytoextraction are often hampered by metal phytotoxicity (Vangronsveld et al., 2009). Therefore, it is crucial to enhance our current understanding of metal-induced stress responses in plants and provide scientific clues to ameliorate phytoextraction strategies.

A recurring cellular response in metal-exposed plants, independent of the species and exposure time, is an increased generation of reactive oxygen species (ROS) such as superoxide (O${}_{2}^{•-}$), hydrogen peroxide (H_2_O_2_) and the hydroxyl radical (^•^OH; Schützendübel and Polle, 2002; Sharma and Dietz, 2009). Under optimal physiological conditions, ROS are constantly produced as by-products of aerobic metabolism in chloroplasts, mitochondria and peroxisomes. However, their production is tightly controlled and maintained at a low level by the antioxidative defense network of plant cells. This system consists of enzymes neutralizing O${}_{2}^{•-}$ and H_2_O_2_ such as superoxide dismutase (SOD), catalase (CAT), peroxidases (POD) and peroxiredoxins (Prx), complemented by metabolites such as ascorbate (AsA) and glutathione (GSH). All subcellular compartments are equipped with specific antioxidative enzymes and metabolites maintaining the cellular redox balance within certain limits (Mittler et al., 2004). However, under abiotic stress conditions such as metal exposure, the equilibrium between ROS production and detoxification is disturbed in favor of the former. While redox-active metals such as Cu and Fe are able to directly generate ROS via Fenton and Haber-Weiss reactions, metals without redox properties (e.g. Cd or Hg) only indirectly contribute to ROS production (Schützendübel and Polle, 2002; Verbruggen et al., 2009).

Whereas ROS are closely linked to hormonal signaling networks in a developmental context (Overmyer et al., 2003; Diaz-Vivancos et al., 2013), it is now widely accepted that they also constitute an ambiguous role during stress responses (Dat et al., 2000). Being toxic molecules, ROS are able to oxidatively injure cells (Møller et al., 2007), but they also regulate defense pathways leading to cellular protection and acclimation (Mittler et al., 2004; Petrov and Van Breusegem, 2012). In addition, recent research also suggests a major role for plant hormones interacting with redox signaling to control adaptive responses to environmental stresses (Mittler et al., 2011; Bartoli et al., 2013; Baxter et al., 2014). More specifically, ethylene has been put forward as an important stress hormone under abiotic stress conditions (Dietz et al., 2010). Therefore, the aim of this review is to highlight our current understanding of the role ethylene plays during metal stress in plants. Experimental evidence for the relationship between ethylene and metal exposure is discussed at the level of ethylene biosynthesis as well as signaling, in which different reports support a link between ethylene and metal tolerance or sensitivity. Finally, special attention is paid to the growing body of evidence suggesting a clear integration between ethylene and the broad network of signaling responses activated in metal-exposed plants.

## Weighing the evidence for a relation between ethylene and metal stress

In the following sections, results of different studies are discussed and point toward a role for ethylene during metal stress responses in plants (Table 1). However, when interpreting these results, it is important to take various aspects related to the experimental design into account. First of all, metal-specific properties should be considered. As discussed before, both essential and non-essential metals cause phytotoxic responses, albeit at different exposure levels. Furthermore, experiments can be conducted using massive or environmentally realistic metal concentrations. Under severe stress conditions, ethylene production might be simply increased by tissue damage and necrosis (Lynch and Brown, 1997). Stress severity will affect the activation of specific signal transduction pathways, for example those related to ethylene (Kacperska, 2004). Although Kacperska (2004) proposed that increased ethylene synthesis is a characteristic feature of the alarm situation during severe stress, it was also observed during exposure to mild and environmentally realistic Cd concentrations (Schellingen et al., 2014). Nonetheless, the extent and consequences of augmented ethylene production should always be interpreted with the applied exposure concentrations in mind (Thao et al., 2015).

**Table 1:** **Metal exposure differentially affects ethylene biosynthesis and signaling in plants**.

**Metal**	**Concentration**	**Exposure time**	**Tissue type**	**Species**	**Observations**	**References**
Al	10 or 50 μM AlCl_3_	24 h	Root apices	*L. japonicus*	↑ ACO activity ↑ ethylene (max after 30 min) Al and cobalt/AVG: ↓ ethylene ↓ inhibition of root elongation	Sun et al., 2007
	10 μM AlCl_3_	2 and 24 h	Root apices	*M. truncatula*	↑*ACS* and *ACO* expression	Sun et al., 2007
	50 μM AlCl_3_	24 h	Root apices	*A. thaliana*	↑ ethylene (max after 30 min)	Sun et al., 2010
			Roots		Al and cobalt/AVG/AgNO_3_: ↓ inhibition of root elongation	
	50 μM AlCl_3_	0.5, 2, and 12 h	Roots	*A. thaliana*	↑*ACS* and *ACO* expression	Sun et al., 2010
As	100 and 200 μM As(V)	1.5 to 3 h	Roots	*A. thaliana*	↑ expression of ethylene-related genes in tolerant Col-0 ecotype *ERF* = As tolerance-associated	Fu et al., 2014
Cd	0.5 mM CdCl_2_	14 h	Leaf discs	*T. aestivum*	↑ ethylene	Groppa et al., 2003
	14, 28 or 42 mg kg^−1^	10 days	Chloroplast membranes	*H. vulgare*	↑ ethylene (14 and 28 mg kg^−1^) ↓ ethylene (42 mg kg^−1^)	Vassilev et al., 2004
	5 or 50 μM CdSO_4_	2, 6, and 30 h	Shoots and roots	*A. thaliana*	↑*ACS* and *ACO* expression (30 h, 50 μM Cd) ↑*ERF* expression (all conditions)	Herbette et al., 2006
	50 μM CdCl_2_	15 days	Roots	*P. sativum*	↑ ethylene	Rodríguez-Serrano et al., 2006
	10 or 50 μM Cd	2 h	Roots	*A. thaliana*	↑*ACS* (50 μM) and *ERF* (10 and 50 μM) expression	Weber et al., 2006
	400 μM CdSO_4_	24 h	Different plant parts	*A. thaliana*	↑ ethylene	Arteca and Arteca, 2007
	0.1 mM CdSO_4_	75 h	Suspension cells	*L. esculentum*	↑ ethylene during the first 24 h Cd and AVG/STS: ↓ cell death	Iakimova et al., 2008
	50 μM CdCl_2_	14 days	Leaves	*P. sativum*	↑ ethylene	Rodríguez-Serrano et al., 2009
	200 mg kg^−1^ CdCl_2_	30 days	Leaves	*B. juncea*	↑ ACS activity ↑ ethylene	Masood et al., 2012
	10 or 25 mg l^−1^CdCl_2_	3, 6, and 24 h	Root tips (RNA) Whole plants (ethylene)	*G. max*	↑*ACS* expression (3 and 6 h) ↑ ethylene	Chmielowska-Bąk et al., 2013
	50 μM CdCl_2_	30 days	Leaves	*B. juncea*	↑ ACS activity ↑ ethylene	Asgher et al., 2014
	5 μM CdCl_2_	15 days	Leaves	*H. vulgare*	↑ ethylene Cd-tolerant genotype: ↑*ACO* expression Cd-sensitive genotype: ↓ ethylene responsive genes	Cao et al., 2014
	5, 10, 25 or 100 μM CdSO_4_	24 and 72 h	Shoots and roots (RNA/ACC) Whole plants (ethylene)	*A. thaliana*	↑*ACS* and *ACO* expression ↑ ACC (free and conjugated) ↑ ethylene ↑ ethylene responsive genes	Schellingen et al., 2014
	50 μM CdCl_2_	3 h	Roots	*O. sativa*	↑*ACO* expression	Trinh et al., 2014
	5 μM CdCl_2_	16 days	Whole plants	*A. thaliana*	↓ ethylene	Carrió-Seguí et al., 2015
	200 mg kg^−1^ CdCl_2_	30 days	Leaves	*T. aestivum*	↑ ACS activity ↑ ethylene	Khan et al., 2015
Cr	200 μM K_2_CrO_4_[Cr(VI)]	1 to 3 h	Roots	*O. sativa*	↑*ACS, ACO* and *EIN3;4* expression	Trinh et al., 2014
Cu	10 mM CuSO_4_	48 h	Leaves	*N. glutinosa*	↑*ACO* expression	Kim et al., 1998
	25, 100 or 500 μM CuSO_4_	7 h	Whole plants	*A. thaliana*	↑ ethylene Cu and AVG: ↓ ethylene	Mertens et al., 1999
	0.5 mM CuCl_2_	14 h	Leaf discs	*H. annuus T. aestivum*	↑ ethylene	Groppa et al., 2003
Cu	10 μM Cu	2 h	Roots	*A. thaliana*	↑*ACS* and *ERF* expression	Weber et al., 2006
	400 μM CuSO_4_	24 h	Different plant parts	*A. thaliana*	↑ ethylene	Arteca and Arteca, 2007
	2.5 mM CuCl_2_	0.5 to 6 h	Whole plants	*B. oleracea*	↑*ACS* and *ACO* expression	Jakubowicz et al., 2010
	25 or 50 μM CuSO_4_	9 days	Whole plants	*A. thaliana*	= ethylene	Lequeux et al., 2010
Fe	200 mg l^−1^ FeSO_4_	24 h	Leaves	*O. sativa*	↑ ethylene	Yamauchi and Peng, 1995
	300 mg l^−1^ FeSO_4_	10 days	Shoots and roots		= ethylene	
	300 mg l^−1^ FeSO_4_	24 h	Leaves of derooted plants		↑ ethylene	
Hg	500 or 1000 μM HgCl_2_	15 days	Roots	*H. vulgare*	↑ expression of ethylene responsive genes	Lopes et al., 2013
	10 μM HgCl_2_	6, 12, 24, and 48 h	Whole plants	*M. truncatula*	Altered expression of ethylene responsive genes	Zhou et al., 2013
	25 μM Hg	1 to 3 h (short)	Root apices	*O. sativa*	↑ expression of *ACS, ACO* and ethylene responsive gene	Chen et al., 2014
		24 h (long)			↑*ACO* expression	
	3 μM HgCl_2_	3, 6, and 24 h	Roots	*M. sativa*	↑ expression of *ACS, ACO* and ethylene responsive genes Hg + 1-MCP: ↓ induction of ethylene- related genes	Montero-Palmero et al., 2014a
Li	0.1, 1, 10 or 50 mM LiCl	2 h	Whole plants	*A. thaliana*	↑*ACS* expression	Liang et al., 1996
	30 mM LiCl	6 days	Leaves	*N. tabacum*	↑ ethylene Li and AVG: ↓ ethylene no necrotic spots	Naranjo et al., 2003
Ni	50, 100, 200, 400 and 800 μM NiSO_4_	24 h	Inflorescence stalks and leaves	*A. thaliana*	= ethylene	Arteca and Arteca, 2007
	200 mg kg^−1^ NiSO_4_	30 days	Leaves	*B. juncea*	↑ ACS activity ↑ ethylene	Khan and Khan, 2014
Pb	500 mg l^−1^ Pb(NO_3_)_2_	12 days	Shoots and roots	*S. drummondii*	↑ expression of a putative *ACS*/*ACO* gene (shoots)	Srivastava et al., 2007
	0.5 mM Pb(NO_3_)_2_	14 days	Whole plants	*A. thaliana*	↑*EIN2* expression	Cao et al., 2009
Zn	25, 100 or 500 μM ZnSO_4_	7 h	Whole plants	*A. thaliana*	↑ ethylene	Mertens et al., 1999
	50, 100, 200, 400 and 800 μM ZnSO_4_	24 h	Inflorescence stalks and leaves	*A. thaliana*	= ethylene	Arteca and Arteca, 2007
	200 mg kg^−1^ ZnSO_4_	30 days	Leaves	*B. juncea*	↑ ACS activity ↑ ethylene	Khan and Khan, 2014

For each study, the experimental setup (metal concentration, exposure time, tissue type and plant species) is shown to facilitate the interpretation of metal-induced responses related to ethylene biosynthesis and the induction of the ethylene signaling cascade. In some studies, the functional role of ethylene during metal stress is studied by inhibiting ethylene biosynthesis using aminoethoxyvinylglycine (AVG) or cobalt, as well as by inhibiting ethylene signaling using 1-methylcyclopropene (1-MCP), silver nitrate (AgNO_3_) or silver thiosulfate (STS).

It is important to discriminate between primary and secondary metal stress-induced events in plants. For example, metal toxicity often leads to nutrient deficiency (Lynch and Brown, 1997; Cuypers et al., 2009), which in its turn is related to alterations in ethylene biosynthesis and signaling (Iqbal et al., 2013a). Furthermore, one of the primary responses of plants to metal stress is the generation of ROS and induction of an oxidative challenge. Redox-active and non-redox-active metals affect the cellular redox state in a different way, which might also influence plant responses related to ethylene as discussed in the section “Interaction between Ethylene and ROS Signaling.” Although some kinetic studies have been conducted (Montero-Palmero et al., 2014a; Schellingen et al., 2014, 2015a,b), more in-depth research is required to decipher the exact order of both primary and secondary events affecting ethylene production under metal stress.

Chelation followed by vacuolar sequestration is a common strategy exploited by plants to maintain low concentrations of free metal(loid)s in the cytosol. Important chelators either contain thiol groups [e.g. metallothioneins, glutathione and phytochelatins (PCs)] or not (e.g. histidine, nicotianamine and organic acids; Seth et al., 2012; Anjum et al., 2015). Especially for GSH, evidence is pointing toward a relationship with ethylene biosynthesis and signaling under metal stress (see section “Crosstalk between Ethylene and GSH”). However, it should be noted that not every metal(loid) is equally connected to this chelating compound (Anjum et al., 2015). Therefore, it is important to consider metal-specific properties when discussing the link between ethylene and GSH.

Finally, different experimental strategies are used to unravel the functional role of ethylene during metal stress. On the one hand, ethylene biosynthesis or signaling can be pharmacologically inhibited. On the other hand, different results can be obtained when studying mutants defective in one or both processes. Furthermore, not all mutations will lead to complete inhibition of ethylene biosynthesis or signaling due to functional redundancy (e.g. different ethylene receptors). Some studies use transformants that overexpress ethylene-related genes, often derived from other plants or even organisms, to study the functional role of ethylene in metal tolerance. Correct data interpretation is therefore only possible when the setup is taken into account (cfr. *infra*; Thao et al., 2015). The studies summarized in this review clearly point toward an intimate relationship between ethylene and metal stress in plants. However, much work remains to be done to finally determine the mechanistic processes underlying this link and apply this knowledge in field conditions, e.g. during phytoremediation.

## Metal stress affects ethylene biosynthesis and signaling at multiple levels

In 1901, the Russian plant physiologist Neljubov reported that etiolated pea plants grew horizontally in the laboratory and upright in outside air (Neljubov, 1901). He attributed this abnormal growth response to ethylene in illuminating gas and is therefore credited with its discovery as biologically active compound (Bleecker and Kende, 2000). It took 33 more years to provide chemical proof that plants indeed synthesize this volatile molecule themselves (Gane, 1934), providing an important indication to investigate the function of ethylene as endogenous signaling molecule. Currently, this simple two-carbon atom molecule (C_2_H_4_) is “all around” and known to be involved in almost all developmental and physiological processes in plants (De Martinis et al., 2015). It triggers senescence, influences growth, leads to various morphogenetic effects and—important within the scope of this review—acts as “stress hormone” in diverse biotic and abiotic stress conditions (Bleecker and Kende, 2000; Lin et al., 2009; Vandenbussche et al., 2012; Van de Poel et al., 2015).

### Ethylene biosynthesis is altered under metal stress

**Figure 1:**
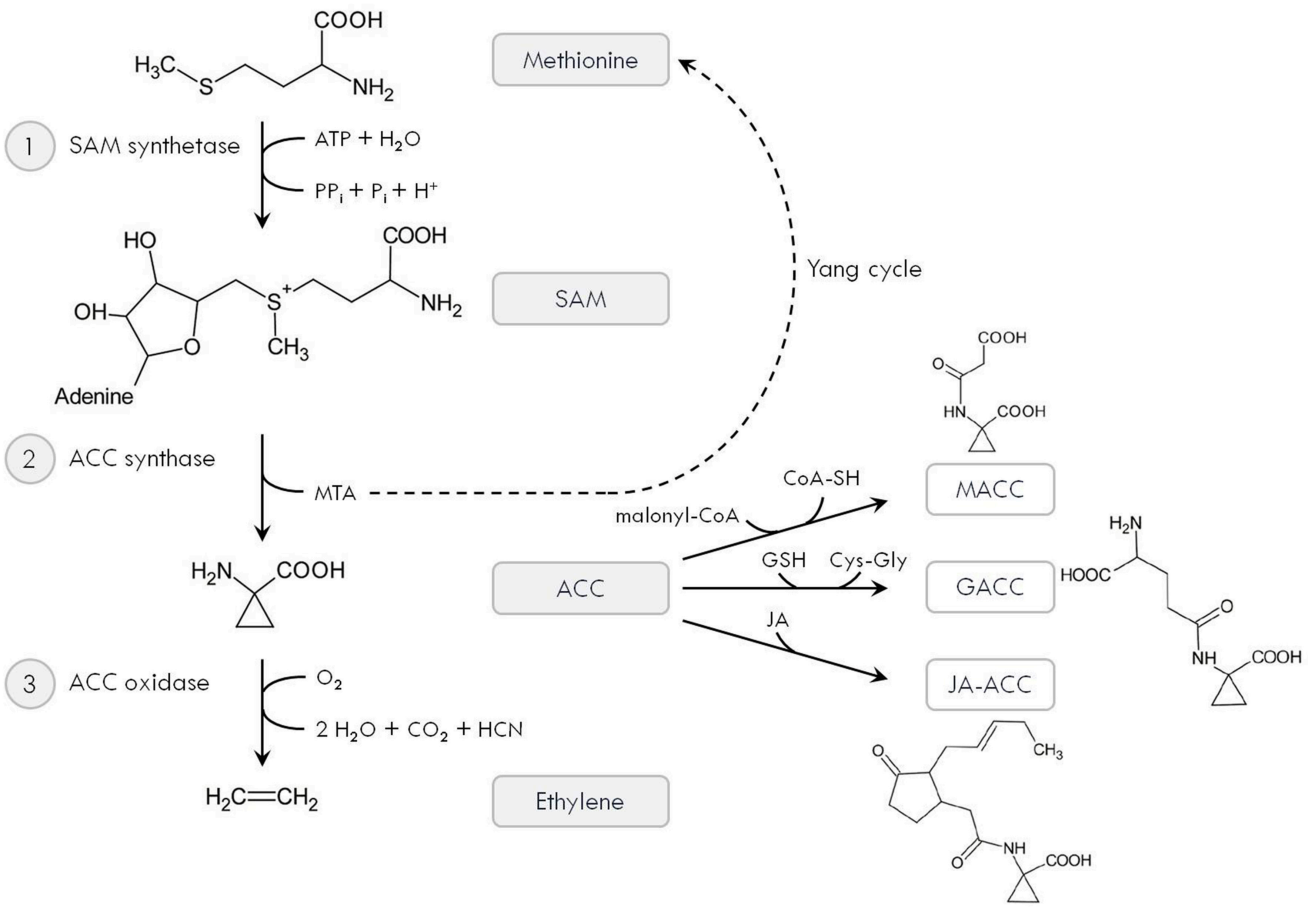
**Ethylene biosynthesis pathway**. The amino acid methionine is converted to S-adenosyl-methionine (SAM) by SAM synthetase (1), which requires ATP. Using SAM as a substrate, 1-aminocyclopropane-1-carboxylic acid (ACC) is produced by ACC synthase (ACS) (2). This also releases 5′-methylthioadenosine (MTA), which is recycled back to methionine via the so-called “Yang cycle.” Finally, ACC is oxidized by ACC oxidase (ACO) (3) to produce ethylene, CO_2_ and cyanide (HCN). In addition, ACC can be converted to its major conjugate 1-malonyl-ACC (MACC) using malonyl-CoA. It can also react with GSH to form γ-glutamyl-ACC (GACC) or with JA to produce jasmonyl-ACC (JA-ACC).

More than 30 years ago, Yang and co-workers elucidated the ethylene biosynthesis pathway, which involves the consecutive action of three enzymes (Figure 1; Yang and Hoffman, 1984). First, the amino acid methionine is converted to S-adenosyl-methionine (SAM) by SAM synthetase. Using SAM as a substrate, 1-aminocyclopropane-1-carboxylic acid (ACC) is produced by ACC synthase (ACS). This is the rate-limiting step in the ethylene biosynthesis pathway and releases 5′-methylthioadenosine (MTA), which is recycled back to methionine via the so-called “Yang cycle.” In the presence of O_2_, ACC is degraded by ACC oxidase (ACO) to produce ethylene, CO_2_ and cyanide (HCN; Figure 1). The latter is detoxified by β-cyanoalanine synthase to prevent toxicity of accumulating HCN at high ethylene biosynthesis rates (Bleecker and Kende, 2000; De Paepe and Van Der Straeten, 2005; Lin et al., 2009).

Of the 12 members of the ACS multigene family in *Arabidopsis thaliana*, eight encode functional ACS enzymes (isoforms 2, 4–9 and 11). While *ACS1* is inactive and *ACS3* encodes a pseudogene, isoforms 10 and 12 encode aminotransferases (Yamagami et al., 2003; Van de Poel and Van Der Straeten, 2014). The complexity of the ACS family is further enhanced at the structural and functional level by the formation of heterodimers. Although individual members of the gene family display specific developmental and physiological roles, significant combinatorial interplay exists between different isoforms. Various internal as well as external stimuli [developmental cues (e.g. senescence and ripening), light, hormones (e.g. auxin, cytokinin and ethylene), biotic (e.g. pathogens), and abiotic (e.g. heat) stress factors] regulate the production of ethylene at the level of *ACS* gene expression (Tsuchisaka et al., 2009; Van de Poel and Van Der Straeten, 2014). For example, *ACS8* transcript levels are controlled by light and shade as well as the circadian clock (Vandenbussche et al., 2003; Thain et al., 2004). Expression of *ACS2* and *ACS6* often appears to be regulated by different stresses such as ozone, salinity and hypoxia (Vahala et al., 1998; Arteca and Arteca, 1999; Peng et al., 2005). In addition, ACS enzymes have a highly variable carboxylic end that serves as a regulatory domain responsible for post-transcriptional regulation. This is due to the presence of mitogen-activated protein kinase (MAPK) and/or calcium-dependent protein kinase (CDPK) target sites, with phosphorylation playing an important role in ACS protein stability (Chae and Kieber, 2005; Yoon and Kieber, 2013).

In the final biosynthetic reaction, ACC is converted to ethylene by ACO. When ethylene production rates are high, for example during post-climacteric ripening of tomato fruit (Van de Poel et al., 2012), ACO can also act rate-limiting in ethylene biosynthesis. It is a ferrous-dependent non-heme oxygenase and uses a single electron from AsA to open the ACC ring (Murphy et al., 2014). In *A. thaliana*, five different *ACO* genes appear to be expressed in all tissues. However, differential accumulation of specific *ACO* transcripts is observed during various physiological processes and environmental conditions (De Paepe and Van Der Straeten, 2005; Argueso et al., 2007; Lin et al., 2009; Ruduś et al., 2012). Several *ACO* genes were shown to be auto-regulated by ethylene (De Paepe et al., 2004) and recently, evidence is suggesting post-transcriptional/translational regulation mechanisms for ACO as well (Dilley et al., 2013; Van de Poel et al., 2014; Van de Poel and Van Der Straeten, 2014).

Instead of being degraded by ACO, ACC can also be converted to its major conjugate 1-malonyl-ACC (MACC) using malonyl-coenzyme-A. Secondly, ACC can react with GSH to form γ-glutamyl-ACC (GACC). Finally, jasmonic acid also forms a conjugate with ACC, producing jasmonyl-ACC (JA-ACC; Figure 1). These conjugates could regulate the pool of available ACC and potentially affect ethylene production. However, the exact molecular and biochemical function of ACC conjugates deserves further investigation, as recent studies report ACC to function as a signal itself (Yoon and Kieber, 2013; Van de Poel and Van Der Straeten, 2014). Increased levels of conjugated ACC were observed in both roots and leaves of Cd-exposed *A. thaliana* plants (Schellingen et al., 2014; Table 1), supporting a role for ACC conjugation during metal stress. Future research should be conducted to reveal the molecular nature of these conjugates. In particular, GACC might be involved as GSH is known to play a central role in defense to metal stress via its chelating, antioxidant and signaling properties (Jozefczak et al., 2012; Hernández et al., 2015).

Several reports have shown that the effects of metal stress on ethylene production in plants are both metal- and concentration-specific (Abeles et al., 1992; Thao et al., 2015; Table 1). It has been suggested that Cd could be the most phytotoxic inorganic ion able to stimulate ethylene production by plants (Abeles et al., 1992; Arteca and Arteca, 2007). Cadmium-induced increases in ethylene production were observed in *Hordeum vulgare* (Vassilev et al., 2004), *Lycopersicon esculentum* (Iakimova et al., 2008), *Pisum sativum* (Rodríguez-Serrano et al., 2006, 2009), *Brassica juncea* (Masood et al., 2012; Asgher et al., 2014), *Glycine max* (Chmielowska-Bąk et al., 2013), *A. thaliana* (Schellingen et al., 2014) and *Triticum aestivum* plants (Khan et al., 2015). On the other hand, long-term (16 days) Cd exposure decreased ethylene release in *A. thaliana* (Carrió-Seguí et al., 2015). Interestingly, a Cd-tolerant *H. vulgare* genotype showed a larger increase in ethylene emission after 15 days of Cd exposure as compared to a Cd-sensitive genotype (Cao et al., 2014). Up to 6 h after exposure to excess Cu or Zn (25–500 μM), seven-days-old *A. thaliana* seedlings grown on hydroponics produced more ethylene than unexposed seedlings (Mertens et al., 1999). In contrast, no significant changes in ethylene emission were detected for *A. thaliana* seedlings *in vitro* grown in the presence of 25 or 50 μM Cu during 9 days (Lequeux et al., 2010), suggesting an effect of exposure time and/or plant age. Excess Cu (500 μM) did induce increased ethylene production in *Helianthus annuus* and *T. aestivum* leaf discs. On the other hand, exposure to 500 μM Cd only enhanced its emission in *T. aestivum* leaves (Groppa et al., 2003), pointing toward species-specific responses to metal stress. Moreover, different *A. thaliana* plant parts showed a various induction of ethylene release after exposure to excess Cu or Cd, with the highest production rate observed in inflorescences. This response declined with increasing age of the different plant parts and did not occur in plants exposed to Ni or Zn (Arteca and Arteca, 2007). Nonetheless, Ni and Zn exposure led to higher ethylene release from *B. juncea* leaves (Khan and Khan, 2014) and aluminum (Al) induced a rapid evolution of ethylene from *Lotus japonicus* (Sun et al., 2007) and *A. thaliana* root apices (Sun et al., 2010). Also Fe (Yamauchi and Peng, 1995) and lithium (Li) toxicity (Naranjo et al., 2003) were reported to be linked to stress-induced ethylene production (Table 1).

Although most studies only investigated the effects of metal exposure on ethylene release by plants, the mechanistic basis is becoming increasingly clear (Table 1). For example, Cu induced an increased expression of *ACO1* and *ACO3* genes in *Nicotiana glutinosa* (Kim et al., 1998). It has been suggested that upregulation of *ACO* genes serves as a good ethylene production indicator (Ruduś et al., 2012). Nevertheless, ACC production by ACS covers the rate-limiting step in the ethylene biosynthesis pathway. Sun et al. (2007) have attributed the induction of ethylene evolution from roots of Al-exposed *L. japonicus* plants to increased ACO activity, but also observed upregulated *ACS* and *ACO* gene expression in *Medicago truncatula* after Al exposure. While Li had a variable effect on *ACS* expression (Liang et al., 1996), Cu highly increased *ACS* transcript levels in *A. thaliana* plants (Weber et al., 2006). Activity of ACS increased in *B. juncea* plants exposed to Cd (Asgher et al., 2014), Ni or Zn (Khan and Khan, 2014), as well as in Cd-exposed *T. aestivum* plants (Khan et al., 2015). Transcript levels of *ACS* and *ACO* genes were rapidly enhanced in Cu-exposed *B. oleracea* (Jakubowicz et al., 2010), Al-exposed *A. thaliana* (Sun et al., 2010), chromium (Cr)-exposed *Oryza sativa* (Trinh et al., 2014), and Hg-treated *O. sativa* (Chen et al., 2014) and *M. sativa* plants (Montero-Palmero et al., 2014a). In addition, Cd was shown to enhance *ACS* gene expression in *G. max* (Chmielowska-Bąk et al., 2013) and *ACS* and/or *ACO* transcription in *H. vulgare* (Cao et al., 2014), *O. sativa* (Trinh et al., 2014) and *A. thaliana* plants (Herbette et al., 2006; Weber et al., 2006; Schellingen et al., 2014; Table 1). In the latter study, the Cd-induced increase in ACC and ethylene biosynthesis was mainly attributed to upregulated *ACS2* and *ACS6* expression, as mutants lacking both isoforms did not show enhanced ethylene release when exposed to Cd (Schellingen et al., 2014). These enzymes are both phosphorylated by the MAPKs MPK3 and MPK6, increasing their half-life (Liu and Zhang, 2004; Joo et al., 2008; Lin et al., 2009; Han et al., 2010; Skottke et al., 2011). Furthermore, MPK3 and MPK6 are able to induce *ACS2* and *ACS6* transcription via the transcription factor WRKY33 (Li et al., 2012). As MAPKs are clearly implicated in metal-induced signaling responses in plants (Opdenakker et al., 2012), they might affect ethylene biosynthesis during metal stress. Finally, whereas most studies focused on *ACS* or *ACO* gene expression levels, Dorling et al. (2011) have pointed out the importance of also examining the effects of metal stress on enzyme abundance, activity and post-translational modifications.

### Ethylene signaling is affected in metal-exposed plants

The ethylene signaling cascade starts with its perception by a family of membrane-bound receptors that are predominantly localized at the endoplasmic reticulum (ER). Because of its volatile nature, ethylene can freely diffuse throughout the cell from the site of production to the ER. In *A. thaliana*, five genes encode a high affinity receptor for ethylene: *ETHYLENE RESISTANT 1* and *2* (*ETR1/2*), *ETHYLENE RESPONSE SENSOR 1* and *2* (*ERS1/2*), and *ETHYLENE INSENSITIVE 4* (*EIN4*). Although some functional specificity exists among the different isoforms, they are largely redundant in controlling the ethylene response in plants (Merchante et al., 2013). In the absence of ethylene, its receptors actively suppress the downstream response (Hua and Meyerowitz, 1998). All receptors possess an N-terminal transmembrane domain to bind ethylene, a domain involved in protein-protein interactions between different receptor types and a C-terminal domain to interact with downstream components of the signaling cascade. A functional receptor unit consists of a homo- or heterodimer able to bind ethylene, although associations of higher order can give rise to receptor clusters in the ER membrane (Merchante et al., 2013). REVERSION TO ETHYLENE SENSITIVITY 1 (RTE1) negatively regulates ethylene responses by specifically activating ETR1 (Resnick et al., 2006, 2008). Furthermore, Cu is required for ethylene binding as well as receptor functionality and is delivered to the receptors by the intracellular RESPONSIVE TO ANTAGONIST 1 (RAN1) Cu transporter (Hirayama et al., 1999). Although the role of Cu in ethylene perception is well established, recent results point toward its involvement in ethylene biosynthesis as well. Indeed, *A. thaliana* plants grown under Cu deficient conditions release less ethylene (Carrió-Seguí et al., 2015).

**Figure 2:**
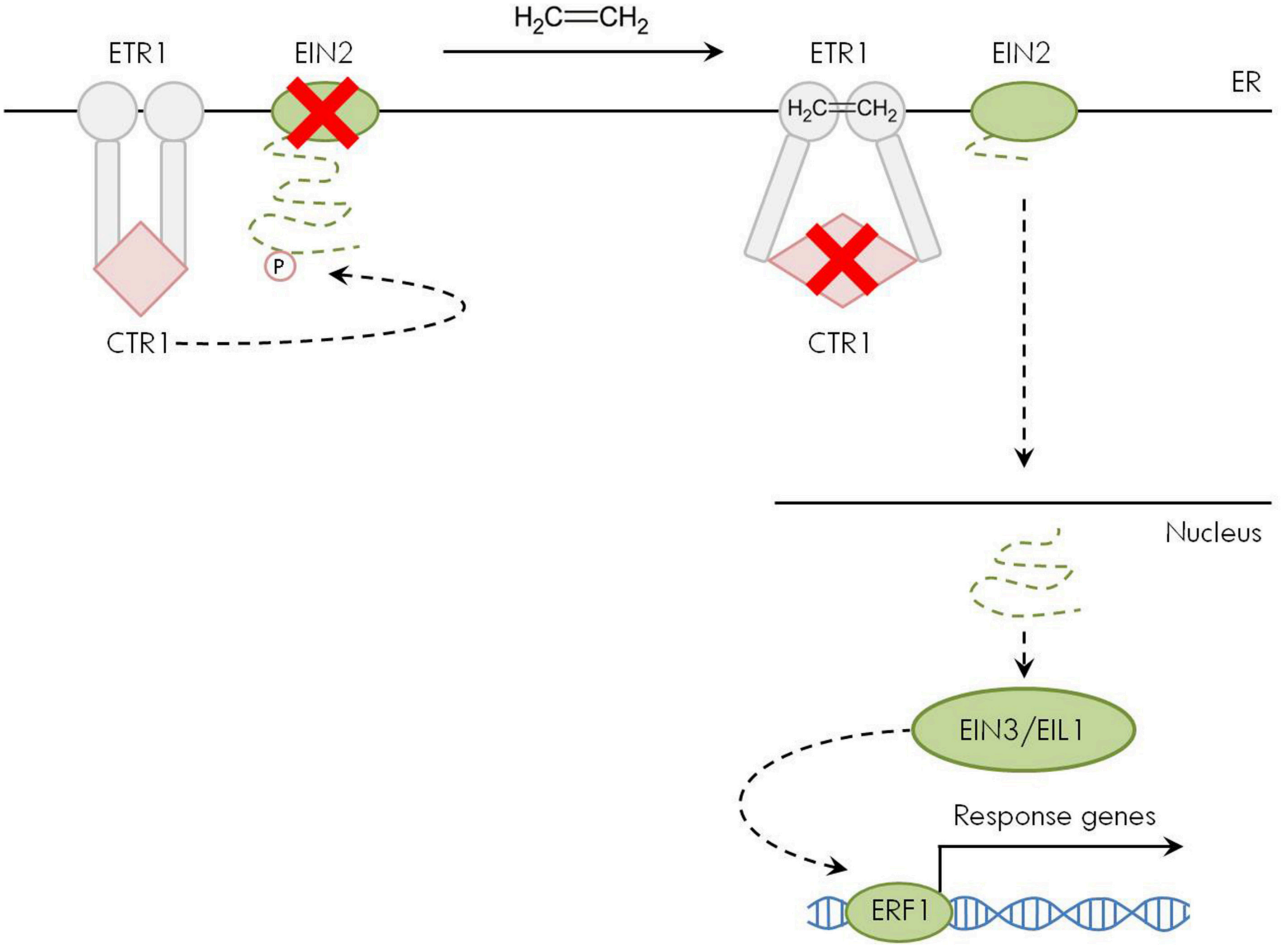
**Ethylene signal transduction pathway**. In the absence of ethylene (left part), the ER-membrane embedded receptors such as ETHYLENE RESISTANT 1 (ETR1) activate CONSTITUTIVE TRIPLE RESPONSE 1 (CTR1). This Raf-like protein kinase phosphorylates ETHYLENE INSENSITIVE 2 (EIN2) at the C-terminal domain, which is thereby inactivated. When ethylene is present (right part), its binding to the receptors inactivates CTR1. The C-terminal domain of EIN2 translocates to the nucleus and activates the downstream signaling cascade via ETHYLENE INSENSITIVE 3 (EIN3)/EIN3-LIKE 1 (EIL1) and ETHYLENE RESPONSIVE FACTOR 1 (ERF1), finally affecting the transcription of ethylene responsive genes.

In the absence of ethylene, the receptors activate a Raf-like protein kinase called CONSTITUTIVE TRIPLE RESPONSE 1 (CTR1), which is a negative regulator of the downstream ethylene signaling cascade (Kieber et al., 1993; Ju et al., 2012). Because of its physical interaction with the ethylene receptors, CTR1 also resides at the ER membrane (Gao et al., 2003). Without ethylene binding to its receptors, CTR1 forms a homodimer and functions as serine/threonine protein kinase to phosphorylate—and thereby inactivate—the downstream molecule ETHYLENE INSENSITIVE 2 (EIN2) (Figure 2; Ju et al., 2012). The EIN2 protein is an essential positive regulator of ethylene signaling. Furthermore, it is the only gene of the ethylene pathway where a loss-of-function mutation leads to complete ethylene insensitivity (Alonso et al., 1999). Similar to CTR1, EIN2 interacts with the ethylene receptors and is therefore localized at the ER membrane (Bisson et al., 2009; Bisson and Groth, 2010).

Upon ethylene binding to its receptors, CTR1 is inactivated (Ju et al., 2012; Shakeel et al., 2015). As a result, EIN2 is released from its inhibition by CTR1 and transduces the signal via its C-terminal end that physically moves from the ER membrane to the nucleus to activate the downstream components ETHYLENE INSENSITIVE 3 (EIN3) and EIN3-LIKE 1 (EIL1). These short-lived transcription factors act as positive regulators of the ethylene signaling pathway and activate target genes such as *ETHYLENE RESPONSIVE FACTOR 1* (*ERF1*) that in turn affect the expression of secondary response genes in the ethylene-dependent transcription cascade (Figure 2; Yoo et al., 2009; Merchante et al., 2013). The above-described linear signaling pathway is subject to feedback regulation and turnover of different signaling components at the mRNA and protein level as described elsewhere (Guo and Ecker, 2003; Qiao et al., 2009; Zhao and Guo, 2011; Merchante et al., 2013). As it has not been described yet if and how metal stress affects these regulatory mechanisms, this paves the way for future research in this area.

Several studies support a role for ethylene signaling in response to different metals, mostly related to *ERF* expression (Table 1). The ERF proteins belong to the APETALA2/ethylene response element binding protein (AP2/EREBP) transcription factor family, which is known to mediate and integrate hormonal and redox signaling pathways during abiotic stress (Dietz et al., 2010). Roots of *A. thaliana* plants exposed to 50 μM Cd for 2 h showed increased expression levels of *ERF1, ERF2* and *ERF5*, while only *ERF1* expression was induced when Cu (10 μM) was applied (Weber et al., 2006). In addition, exposure to 5 or 10 μM Cd induced expression of *ERF1, ETR2* and *ACO2* in roots and leaves of *A. thaliana* plants after 24 and 72 h (Schellingen et al., 2014). Expression of *ERF2* and *ERF5* was increased in *A. thaliana* roots and shoots after 2, 6 and 30 h exposure to 5 or 50 μM Cd (Herbette et al., 2006). Recently, a whole-genome transcriptional profile from *M. sativa* seedlings exposed to 3 μM Hg for 3, 6 and 24 h demonstrated significant upregulation of several ethylene-responsive genes such as *ERF1*, mostly during the earliest hours of exposure (Montero-Palmero et al., 2014a). Similarly, Hg exposure affected genes related to ethylene signaling in *M. truncatula* (Zhou et al., 2013), *O. sativa* (Chen et al., 2014), *H. vulgare* plants (Lopes et al., 2013). Furthermore, roots of *O. sativa* plants exposed to 200 μM Cr for up to 3 h showed an increased expression of the *EIN3;4* gene (Trinh et al., 2014), while the *EIN2* gene was induced in Pb-exposed *A. thaliana* plants (Cao et al., 2009; Table 1). It is clear from these studies that ethylene signaling is involved in the response of plants to toxic metals (Montero-Palmero et al., 2014b).

## Ethylene is a key regulator of plant responses to metal stress

Various reports discuss the potential implication of ethylene in plant adaptation or tolerance to toxic metals, and plant genotypes emitting more ethylene were suggested to be more metal resistant than those that release less (Lu and Kirkham, 1991). Moreover, the Pb-hyperaccumulator *Sesbania drummondii* showed increasing mRNA levels of a putative *ACS/ACO* gene upon exposure to Pb (Srivastava et al., 2007). Recently, Fu et al. (2014) conducted a transcriptome profiling of genes and pathways associated with As tolerance and toxicity in two *A. thaliana* ecotypes. In the more tolerant Columbia ecotype, genes encoding components of the ethylene signaling pathway were significantly enriched after short-term As exposure as compared to the sensitive Wassilewskija ecotype (Fu et al., 2014). Similarly, Cao et al. (2014) suggested that Cd tolerance in *H. vulgare* is related to the induction of ethylene signaling. Transgenic *N. tabacum* plants overexpressing an *ERF* gene from *Lycium chinense* displayed greater tolerance to Cd stress than non-transformed plants (Guan et al., 2015). On the other hand, ethylene insensitive *etr1-1* and *ein3-3 A. thaliana* mutants were shown to be less sensitive to Li than WT plants (Bueso et al., 2007). These apparent conflicting results can be attributed to metal-specific properties, but are definitely related to the chosen experimental setup as discussed before (metal concentration, exposure time, plant species; Table 1, see section “Weighing the Evidence for a Relation between Ethylene and Metal Stress”). Nevertheless, still little is known about the underlying mechanisms of ethylene regulating plant responses to metal stress and potentially affecting sensitivity vs. tolerance (Asgher et al., 2015).

Mutants defective in ethylene biosynthesis and signaling, together with pharmacological compounds to induce or inhibit these processes, have provided an elegant framework to further unravel the involvement of ethylene in plant metal stress responses. In this way, it was shown that ethylene signaling plays an important role during Cd-induced cell death in cultured tomato cells. Exposure to CdSO_4_ induced rapid cell death and a transiently increased ethylene production within 24 h. Addition of aminoethoxyvinylglycine (AVG) to inhibit ethylene biosynthesis or silver thiosulfate (STS) to block the ethylene receptor led to a marked decrease in Cd-induced cell death (Iakimova et al., 2008). A similar inhibitory effect of AVG was observed during Al-induced cell death in tomato suspension cells (Yakimova et al., 2007).

Using the ethylene-insensitive *Never ripe (Nr)* tomato mutant, ethylene was demonstrated to be involved in Cd-induced lipid peroxidation in roots, leaves and fruits (Gratão et al., 2012). Mutant *A. thaliana* plants without functional *ACS2* and *ACS6* enzymes did not show an increased ethylene release upon short-term (24 to 72 h) exposure to 5 or 10 μM Cd as compared to wild-type (WT) plants. Moreover, Cd-induced decreases in leaf fresh weight were less pronounced in mutants than in WT plants, pointing to a lower Cd sensitivity in the absence of ACS2/6 (Schellingen et al., 2014, 2015a). After prolonged exposure to the same Cd concentrations however, WT and *acs2-1/6-1* knockout mutants were equally sensitive, suggesting an early and transient role for ethylene in Cd-induced stress responses (Schellingen et al., 2015a).

Ethylene insensitive *ein2-1* mutants are more sensitive to Pb (Cao et al., 2009). This was attributed to an increased uptake of Pb and a diminished GSH content (Cao et al., 2009), revealing crosstalk between ethylene and the biosynthesis of this antioxidant and metal chelating compound. Also other studies link ethylene to the metal-induced oxidative stress response (Sun and Guo, 2013; Zhang et al., 2014; Montero-Palmero et al., 2014a; Schellingen et al., 2015a,b), as is discussed in the next section. These results clearly point toward the potential benefit of altering ethylene biosynthesis and/or signaling in future phytoremediation strategies (Montero-Palmero et al., 2014a). This is also supported by the fact that bacteria producing ACC deaminase (ACD) and thereby diminishing ethylene levels in their host plant, have been successfully used in laboratory and field conditions to protect plants from growth inhibition by elements such as As, Cd, Cu, Ni, Pb, and Zn (reviewed by Glick et al., 2007). This enzyme converts the ethylene precursor ACC into α-ketobutyrate and ammonia, which is subsequently used as nitrogen source by the bacteria. This reduces deleterious ethylene levels *in planta* and alleviates the associated stress symptoms (Arshad et al., 2007; DalCorso et al., 2013; Glick, 2014). However, it must be emphasized that the beneficial effects of plant-associated bacteria are also related to the increased availability of nutrients such as P and Fe, the production of phytohormones such as auxins and cytokinins and their competition with phytopathogens that could negatively affect plant health and growth (Weyens et al., 2009). Nonetheless, diminishing ethylene levels seems a promising path to explore, as transgenic plants expressing a bacterial ACD gene display a more resistant phenotype than non-transformed plants when exposed to different metals (Arshad et al., 2007; Glick et al., 2007). It has even been shown that plants possess ACD activity themselves (McDonnell et al., 2009), an intriguing asset which could also be exploited in phytoremediation of metal-polluted soils. However, ethylene production and signaling might also be a beneficial part of metal stress responses in plants (Cao et al., 2014; Fu et al., 2014; Thao et al., 2015). Indeed, ethylene can promote as well as inhibit plant growth (Pierik et al., 2006). Therefore, much work remains to be done prior to altering the ethylene response and improving phytoremediation of metal-contaminated soils.

With regard to plant growth in metal-polluted areas, the root architecture is of great importance. Interestingly, ethylene modulates local and systemic responses to low phosphate (Pi), thereby contributing to the remodeling of the root system architecture to increase Pi uptake (Nagarajan and Smith, 2011). As the root system of plants exposed to toxic metals is also drastically changed (Remans et al., 2012), this opens the window to study the potential involvement of ethylene in this response specifically (De Smet et al., 2015). For example, WT *A. thaliana* plants exposed to increasing Cd concentrations showed a higher lateral root density, which was abolished at higher exposure concentrations. In contrast, the Cd-induced increase in lateral root density was maintained at these higher exposure levels in ethylene insensitive *ein3-1* mutants. Ethylene might therefore modulate lateral root outgrowth during high Cd exposure (Remans et al., 2012). Furthermore, ethylene is implicated in the development of root hairs in Cd-exposed *B. napus* seedlings, as the use of the ethylene biosynthesis inhibitors cobalt chloride (CoCl_2_) and aminooxyacetic acid (AOA) attenuated the Cd-mediated increase in root hair density (Sun and Guo, 2013). Ethylene may also inhibit primary root growth during the early response to Hg, as roots of *M. sativa* seedlings exposed to the ethylene receptor inhibitor 1-methylcyclopropene (1-MCP) as well as roots of ethylene insensitive *ein2-5 A. thaliana* mutants grew more in the presence of moderate Hg concentrations as compared to their untreated or WT counterparts (Montero-Palmero et al., 2014a). In addition, ethylene insensitive *etr1-3* and *ein2-1 A. thaliana* plants were more tolerant to Al stress, as root elongation of both mutants was less inhibited than in WT plants (Sun et al., 2010). For *ein2-1*, root and leaf growth was also less compromised as compared to the WT after 14 days of Al exposure (Zhang et al., 2014). Similarly, root elongation of Al-exposed *A. thaliana* plants was less affected in the presence of antagonists of ethylene biosynthesis (AVG and CoCl_2_) and perception [silver nitrate (AgNO_3_); Sun et al., 2010]. Recently, it was shown that ethylene negatively regulates Al-induced efflux of malate anions in wheat. As malate forms extracellular complexes with Al, this explains the increased Al tolerance observed in ethylene insensitive genotypes (Tian et al., 2014). Again, the potential benefits of ethylene able to reduce root and plant growth during metal stress should not be ignored when interpreting the above-mentioned results.

## Crosstalk between ethylene and other players in the metal stress network

**Figure 3:**
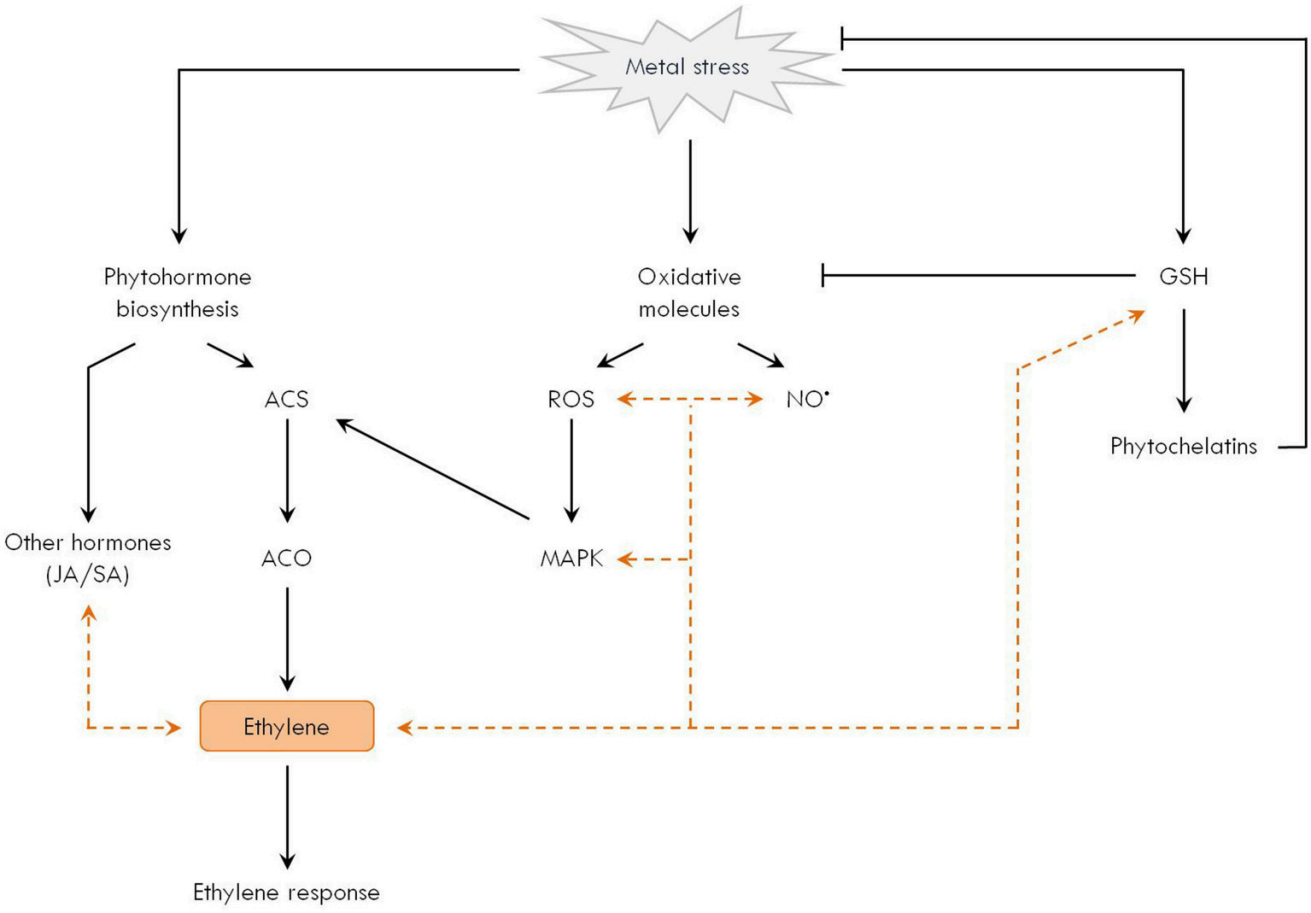
**Ethylene participates in the network of metal-induced signaling responses in plants**. Different signaling pathways are affected by metal exposure in plants. (1) Phytohormones such as ethylene, jasmonic acid (JA) and salicylic acid (SA) are influenced by metal stress. In particular, ethylene biosynthesis is generally activated at the level of ACC synthase (ACS) and oxidase (ACO), thereby stimulating the ethylene signaling cascade. (2) Increased generation of reactive oxygen species (ROS, e.g. H_2_O_2_) and reactive nitrogen species (e.g. NO^•^) sets oxidative signaling pathways in motion, for example those mediated by mitogen-activated protein kinases (MAPK). (3) Glutathione (GSH) is a central player in the metal-induced stress network, not only because of its antioxidant function, but also as a precursor for metal-chelating phytochelatins. It is increasingly clear that these individual players integrate and interact within a broad signaling network in metal-exposed plants. Direct interaction between oxidative stress and ethylene biosynthesis is demonstrated by the MAPK-mediated activation of ACS. In addition, ethylene is shown to affect other players such as JA, SA, ROS, NO^•^, MAPK, and the GSH metabolism as well (indicated by orange dashed arrows).

Research over the past years points toward an intimate interaction between ethylene and other signaling components implicated in the response of plants to metal stress (Figure 3). In the following sections, the experimental evidence is summarized. Nonetheless, it must be emphasized that our knowledge is still scarce, revealing the need for future research to obtain an integrated picture and potentially apply this information in strategies to cope with phytotoxic metals (Thao et al., 2015).

### Interaction between ethylene and ROS signaling

It is widely accepted that ROS act as signaling molecules in abiotic stress responses, interacting with other signaling pathways in a spatiotemporal manner (Bartoli et al., 2013; Baxter et al., 2014). Oxidative stress characterized by an imbalance between ROS and antioxidants in favor of the former is a recurrent response of metal-exposed plants (Sharma and Dietz, 2009), which triggers downstream responses potentially leading to acclimation. Furthermore, it is increasingly clear that signals related to an increased ROS generation are linked to hormonal signaling pathways (Fujita et al., 2006; Baxter et al., 2014). Different studies demonstrated the involvement of ethylene in the stress-induced oxidative burst, as reviewed by Steffens (2014) during salinity, flooding and metal stress responses in *O. sativa*. When ethylene production or perception was inhibited by AVG or STS, respectively, camptothecin-induced H_2_O_2_ production was blocked in *L. esculentum* suspension cells (de Jong et al., 2002). As compared to WT plants, ethylene insensitive *ein2-1 A. thaliana* plants produced less H_2_O_2_ and showed a lower O${}_{2}^{•-}$ production rate when exposed to paraquat. Consequently, mutant seedlings had a lower increase in malondialdehyde (MDA) content, which suggests less oxidative damage compared to the WT (Cao et al., 2006). Similarly, H_2_O_2_ production and MDA content were lower in *ein2-1* as compared to WT plants after Al exposure (Zhang et al., 2014). Furthermore, application of the ethylene receptor blocker STS significantly reduced the H_2_O_2_ content in roots of Cd-exposed *Phaseolus coccineus* plants after 1 and 2 h (Maksymiec, 2011). Cadmium-induced production of O${}_{2}^{•-}$ at the growing root hair tips of *B. napus* was blocked by the ethylene biosynthesis inhibitor AOA, suggesting that ethylene signaling acts upstream of O${}_{2}^{•-}$ (Sun and Guo, 2013). Finally, *A. thaliana cat2-1* mutants that accumulate more H_2_O_2_ under normal growth conditions were more tolerant to Li, although they took up more Li as compared to WT plants. Lithium-exposed *cat2-1* mutants produced less ethylene and showed less induction of ethylene responsive genes than the WT. Therefore, the authors attributed the increased Li tolerance of *cat2-1* mutants to a reduced ethylene production and sensitivity (Bueso et al., 2007).

These results suggest an interaction between ethylene and the ROS network of plants, with ethylene able to affect ROS producing as well as scavenging enzymes and metabolites. The ROS producing NADPH oxidases [also known as respiratory burst oxidase homologs (RBOH)] are put forward as critical signaling hubs in the response of plants to environmental stimuli (Suzuki et al., 2011) such as metal exposure (Remans et al., 2010). Ethylene is an important upstream regulator of O${}_{2}^{•-}$-producing NADPH oxidases (Chae and Lee, 2001), with a regulatory interaction between the ethylene biosynthesis gene *ACS1* and *RBOHD/F* transcription in *B. oleracea* seedlings (Jakubowicz et al., 2010). In *Ipomoea batatas*, the NADPH oxidase inhibitor diphenyleneiodonium decreased ROS production induced by ethephon, an ethylene releasing compound (Chen et al., 2013). Furthermore, ethylene seems to stimulate the apoplastic release of H_2_O_2_ by activating NADPH oxidase isoform D (RBOHD) during biotic stress, as flagellin (flg22)-induced ROS generation diminished in ethylene insensitive *etr1-1* and *ein2-1 A. thaliana* mutants as compared to the WT (Mersmann et al., 2010). Recent reports also indicate a relationship between ethylene and NADPH oxidase during metal stress. For example, inhibition of the ethylene receptors by 1-MCP reduced or even abolished the increase in extracellular H_2_O_2_ production and NADPH oxidase activity observed during the first 6 h of Hg exposure in *M. sativa* root segments (Montero-Palmero et al., 2014a). In addition, Hg-exposed ethylene insensitive *ein2-5* mutants produced less H_2_O_2_ as compared to their WT counterparts (Montero-Palmero et al., 2014a). Upon Cd exposure, expression of *RBOHC* did not increase to WT levels in leaves of *acs2-1/6-1* knockout, *ein2-1*, and *ein2-5* mutant *A. thaliana* plants (Keunen et al., 2015), again supporting a link between ethylene and ROS production by NADPH oxidases during metal stress. Furthermore, ethylene signaling was also related to the transcriptional induction of ALTERNATIVE OXIDASE 1a/d (AOX1a/d), which was lower in leaves of Cd-exposed *ein2-1* and *ein2-5* mutants as compared to WT plants. This enzyme regulates ROS levels and is suggested to modulate the Cd-induced oxidative challenge in *A. thaliana*, requiring ethylene—either directly or indirectly via RBOHC—to be fully induced at the transcript level (Keunen et al., 2015). In line with this, AOX was demonstrated to be involved in ethylene-induced plant cell death as well (Lei et al., 2003).

On the other hand, ethylene might affect the plant's antioxidative defense network as shown by Cao et al. (2006). They demonstrated a constitutively higher transcription of *Cu/Zn SOD 2* (*CSD2*) and *CAT3* genes, leading to enhanced SOD and CAT enzyme activities in *ein2-1* mutants as compared to WT plants. This was also shown in Al-exposed *ein2-1* mutants, which showed differential responses at the level of SOD and CAT activities compared to WT plants (Zhang et al., 2014). The interaction between ethylene and antioxidative defense is further underlined by the fact that *ein2-1, ein3-1* and *ein4* mutant *A. thaliana* plants have a higher AsA content in leaves (Gergoff et al., 2010), which was also observed in ethylene insensitive *Nr* tomato fruits (Alba et al., 2005). Conversely, the *ctr1-1* mutant with a loss-of-function of the negative regulator CTR1 displayed lower leaf AsA levels (Gergoff et al., 2010). Concurrently, ethylene signaling was reported to suppress AsA synthesis and accumulation in tomato leaves (Mazorra Morales et al., 2014). In this regard, it is noteworthy to mention that ethylene biosynthesis requires AsA in the final step (cfr. *supra*), further supporting crosstalk between both compounds. Finally, mutants defective in ethylene perception (*etr1-1*) as well as those overproducing ethylene (*eto1-1*) showed up to five-fold higher α-tocopherol levels during leaf aging. Furthermore, ethylene insensitive *ein3-1* mutants showed a delayed increase in α-tocopherol during water stress (Cela et al., 2009). This antioxidant compound was shown to be essential for the tolerance of *A. thaliana* plants to metal-induced oxidative stress (Collin et al., 2008). Therefore, the interaction between ethylene and the antioxidative defense network mounted during metal exposure can ultimately affect responses leading to sensitivity or tolerance and deserves further investigation. In particular, a relation between ethylene and GSH is suggested and is discussed in a separate section.

Besides ROS, plant cells also produce reactive nitrogen species (RNS) such as nitric oxide (NO^•^) during abiotic stress (Corpas et al., 2011) such as Cd exposure (Arasimowicz-Jelonek et al., 2011; Chmielowska-Bąk et al., 2014). However, the functional role of NO^•^ in metal-challenged plants is not yet fully understood. Different results point toward an interaction between NO^•^ and ethylene, as recently reviewed by Mur et al. (2013). During salt stress, NO^•^ and ethylene were shown to cooperate in the modulation of ion homeostasis. Salt stress-induced NO^•^ production greatly enhanced ethylene emission in *A. thaliana* callus. In its turn, ethylene stimulated the expression of plasma membrane H^+^-ATPase genes, which has been suggested to facilitate Na^+^ efflux into the apoplast and attenuate Na^+^ toxicity under saline conditions (Wang et al., 2009). Such regulatory interactions between NO^•^ and ethylene might also be involved in the response of plants to metal stress. Interestingly, both ethylene and NO^•^ are involved in the upregulation of key genes related to Fe acquisition and homeostasis in *A. thaliana* (García et al., 2010). Iron deficiency is a well-known consequence of metal toxicity [e.g. Cd (Xu et al., 2015)] and seems to increase ethylene sensitivity (García et al., 2010), which potentially affects metal-induced responses related to ethylene as well. It has been shown that nutrient stress—either a reduced or increased availability—affects ethylene biosynthesis and perception in plants via the induction of an oxidative burst (Iqbal et al., 2013a), again highlighting the link between ethylene and ROS signaling.

### Crosstalk between ethylene and GSH

**Figure 4:**
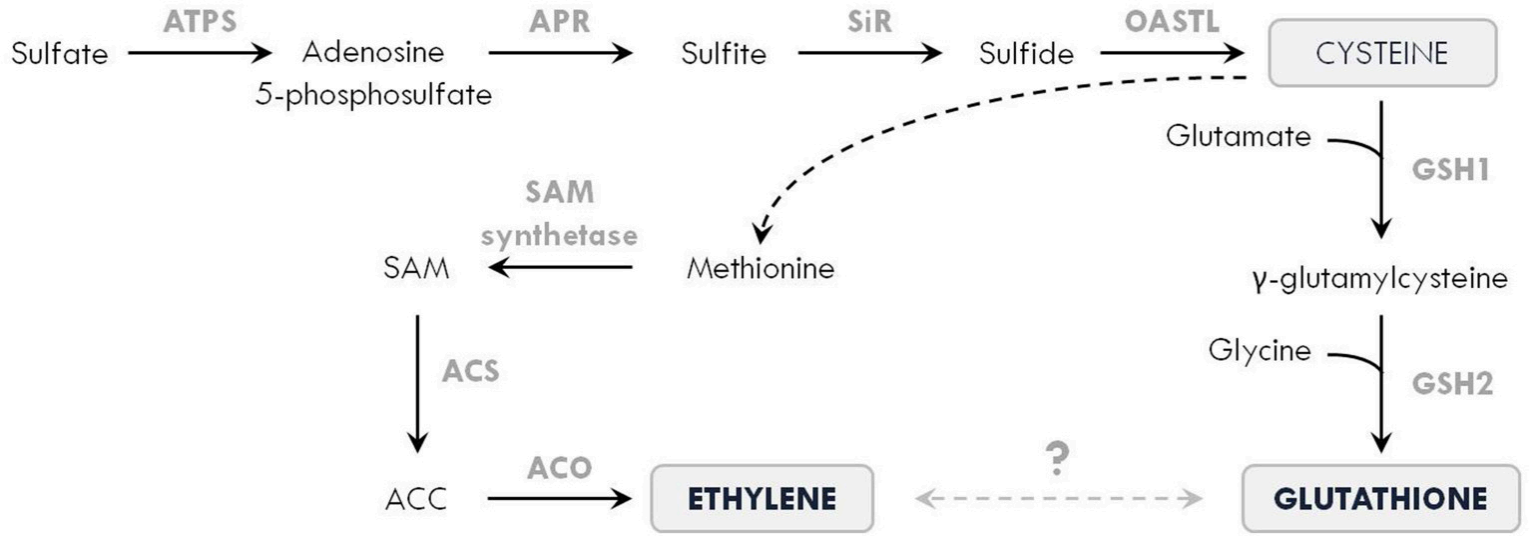
**Simplified scheme of the interaction between sulfur assimilation, ethylene and glutathione biosynthesis in plants**. Sulfur is taken up from the soil as sulfate, which is converted into adenosine 5-phosphosulfate by the enzyme ATP sulfurylase (ATPS). This is further reduced by adenosine 5-phosphosulfate reductase (APR) into sulfite, which is subsequently reduced into sulfide by sulfite reductase (SiR). The enzyme O-acetylserine (thiol) lyase (OASTL) produces cysteine, which is one of the three building blocks that make up glutathione. During glutathione biosynthesis, cysteine is coupled to glutamate by γ-glutamylcysteine synthetase (GSH1) to form γ-glutamylcysteine. In the next step, glycine is added by glutathione synthetase (GSH2) to finally produce glutathione. In addition, cysteine is also required for ethylene formation, as methionine is derived from cysteine via different reactions (depicted by the dashed arrow). In the ethylene biosynthetic pathway, methionine is converted to S-adenosyl-methionine (SAM) by SAM synthetase. In the next steps, 1-aminocyclopropane-1-carboxylic acid (ACC) is produced by ACC synthase (ACS) and subsequently oxidized by ACC oxidase (ACO) to form ethylene (see Figure 1). As ethylene and glutathione fulfill important functions in metal-exposed plants, a trade-off between both might lie at the heart of their interaction regulating plant responses to metal stress.

Tolerance to toxic metals is highly dependent on the metabolism of GSH, a widely distributed biothiol tripeptide (γ-glutamyl-cysteinyl-glycine) in plant cells. As metal chelator, antioxidant and signaling compound, GSH is a key player in metal-induced oxidative stress defenses (Seth et al., 2012). This multifunctional role is related to the sulfhydryl group in cysteine, which has a high affinity toward metals such as Cd and Hg. Furthermore, GSH is the precursor molecule for the synthesis of phytochelatins (PCs), which consist of 2 to 11 GSH molecules and limit the cellular concentration of free metal ions (Jozefczak et al., 2012; Hernández et al., 2015). Using ethephon, it was shown that ethylene induces the activity of ATP sulfurylase (ATPS), leading to an accumulation of sulfur (S) in *B. juncea* (Iqbal et al., 2012). Recently, Iqbal et al. (2013b) reviewed the crosstalk between S assimilation and ethylene signaling in plants. Since GSH synthesis is affected by S availability to produce the amino acid cysteine, ethylene might modulate this process in order to meet the increasing demands for GSH during metal stress. In this regard, it is important to mention that ethylene synthesis itself also requires cysteine to ultimately produce SAM (Figure 4).

Crosstalk between ethylene and GSH is suggested by the observed upregulation of different genes encoding ERF transcription factors in the severe GSH deficient *root meristemless 1-1* (*rml1-1*) *A. thaliana* mutants as compared to WT plants (Schnaubelt et al., 2015). On the other hand, *ERF2* expression was significantly repressed in *cadmium sensitive 2-1* (*cad2-1*) *A. thaliana* mutants that also have lower GSH levels than WT plants, but not as low as those of *rml1-1* mutants (Han et al., 2013). Transgenic *N. tabacum* plants with an enhanced GSH content showed induced ethylene biosynthesis (*ACO*) and signaling (*ERF*) genes (Ghanta et al., 2014). The potential interplay between GSH and ethylene is further underlined by the results of Chen et al. (2013), who demonstrated that exogenous GSH mitigated the ethephon-induced increase in ROS production in sweet potato. Furthermore, ethylene was suggested to increase *de novo* biosynthesis of GSH in ozone-exposed *A. thaliana* plants, thereby protecting against leaf injury (Yoshida et al., 2009).

Increasing evidence points toward a close relationship between ethylene and GSH metabolism during metal stress. For example, ethephon treatment increased GSH levels in Cd-exposed *B. juncea* (Masood et al., 2012). Similarly, GSH levels in Ni- and Zn-exposed *B. juncea* plants were higher after ethephon application, which alleviated metal toxicity (Khan and Khan, 2014). While ACS activity and ethylene production decreased, GSH levels increased in Cd-exposed *T. aestivum* supplied with S (Khan et al., 2015). Transcript levels of genes encoding GSH biosynthesis enzymes were significantly less upregulated, concomitantly with lower GSH levels in leaves of Cd-exposed *acs2-1/6-1* knockout vs. WT *A. thaliana* plants. Therefore, increased ethylene biosynthesis upon Cd exposure seems crucial to mount effective defense responses related to GSH (Schellingen et al., 2015a). In addition, ethylene signaling is implicated in the accumulation of GSH in Al-exposed *A. thaliana* (Zhang et al., 2014), Cd-exposed *L. chinense* (Guan et al., 2015), and *A. thaliana* plants (Schellingen et al., 2015b). The increased Cd tolerance of transgenic tobacco plants overexpressing an *ERF* gene from *L. chinense* was related to an enhanced expression level of GSH biosynthesis genes (Guan et al., 2015). Similar results were obtained in Pb-exposed *A. thaliana* plants, where EIN2 is indispensable to confer metal resistance partially by increasing GSH levels (Cao et al., 2009). These results confirm the suggested interplay between ethylene and GSH in determining metal tolerance vs. sensitivity and open the window to future experiments exploiting this relationship in phytoremediation strategies (Hernández et al., 2015). As discussed before, it should be kept in mind that not all metal(loid)s are equally strong inducers of PC synthesis (Anjum et al., 2015) and will therefore differentially affect GSH levels in plants.

### Interaction between ethylene and MAPK signaling pathways

A signaling pathway linked to ethylene and worthwhile to discuss in the light of metal stress is the ROS-induced MAPK cascade. These kinases are activated at transcript and activity level in different plant species exposed to metals. Furthermore, they interfere with hormone biosynthesis and signaling to activate downstream responses (Opdenakker et al., 2012). As mentioned before, the stress-responsive MPK3 and MPK6 isoforms increase ethylene production by affecting *ACS2* and *ACS6* transcription as well as protein stability (Liu and Zhang, 2004; Li et al., 2012). In addition, MAPK kinase 9 (MKK9) was shown to activate the MPK3/MPK6 cascade and stimulate ethylene biosynthesis in *A. thaliana* (Xu et al., 2008). Other studies have indicated that MAPKs could be involved in ethylene signaling as well (Ouaked et al., 2003; Hahn and Harter, 2009). This might come as no surprise since the negative regulator of ethylene signaling, CTR1, shows sequence similarities with Raf protein kinases and has been presumed to function as a MAPK kinase kinase (MAPKKK). However, no conclusive CTR1-targeted kinases have been identified yet (Zhao and Guo, 2011; Ju and Chang, 2012; Merchante et al., 2013). Nonetheless, nuclear EIN3 was shown to be regulated not only by CTR1 but also by a novel MAPK cascade mediated by MKK9 and MPK3/6 in *A. thaliana*. This cascade functions downstream of CTR1, is activated when ethylene binds to its receptors and stabilizes EIN3 by phosphorylation (Yoo et al., 2008). Recently, Schellingen et al. (2015b) proposed a model where MPK3/6 link ROS production and ethylene signaling during Cd stress in *A. thaliana* leaves. In this model, Cd exposure activates NADPH oxidases, which produce ROS that are sensed by the oxidative signal-inducible kinase1 (OXI1). This kinase then activates MPK3/6, both affecting ACS2/6 enzymes at multiple levels (Schellingen et al., 2015b). Furthermore, Liu et al. (2010) have shown that pretreatment with GSH reduced the activation of MPK3 and MPK6 under Cd stress in *A. thaliana*. This suggests that ROS are involved in Cd-induced MAPK signaling, with a relation to ethylene as both MPK3/6 are able to affect ethylene biosynthesis enzymes (Thao et al., 2015). Therefore, the potential implication of MAPK signaling and its relation with ethylene biosynthesis and/or signaling during metal stress should be explored in more detail.

### Phytohormone crosstalk during metal stress

Interactions between various phytohormones are required to integrate environmental signals and stress tolerance responses (De Paepe and Van Der Straeten, 2005; Kohli et al., 2013). In addition to ethylene, jasmonic acid (JA) and salicylic acid (SA) are mostly implicated in plant stress responses (Van de Poel et al., 2015) such as those mounted during metal exposure (De Smet et al., 2015). Different genes involved in ethylene and JA biosynthesis as well as genes responsive to these hormones were differentially expressed after Hg exposure in *M. sativa, M. truncatula* and *H. vulgare* plants (Montero-Palmero et al., 2014b). Gene expression profiling in Cr-stressed *O. sativa* roots indicated activation of ethylene and JA signaling pathways (Trinh et al., 2014). Furthermore, JA levels rapidly increased in *A. thaliana* and *P. coccineus* plants exposed to Cd or Cu (Maksymiec et al., 2005). From a signaling point of view, JA was shown to trigger ROS production in short-term Cd- and Cu-exposed *A. thaliana* plants, as inhibiting JA biosynthesis by propyl gallate decreased O${}_{2}^{•-}$ and H_2_O_2_ levels after metal exposure (Maksymiec and Krupa, 2006).

A mutual relationship exists between ethylene and JA signaling (Song et al., 2014), which might ultimately affect metal stress responses as well. For example, it has been shown that the JA receptor CORONATINE INSENSITIVE 1 (COI1) is implicated in the inhibition of *Arabidopsis* root growth mediated by ethylene in the light (Adams and Turner, 2010). Furthermore, ethylene insensitive *ein2-1* mutants become ethylene responsive by reducing JA levels via a genetic or chemical approach (Kim et al., 2013). Crosstalk between ethylene and SA during metal stress was supported by the results of Zhang et al. (2014). They showed that *Arabidopsis* mutants insensitive to ethylene (*ein2-1*) or SA [*nonexpressor of pathogenesis-related proteins 1-1* (*npr1-1*)] were more tolerant to Al exposure as compared to WT plants. However, *ein2-1*/*npr1-1* double mutants were less tolerant than WT plants, indicating that the tolerant phenotype of *ein2-1* and *npr1-1* single mutants depended on remaining NPR or EIN function, respectively (Zhang et al., 2014). These results further support studying the complex interaction between various hormonal signaling pathways mediating metal stress responses in plants.

As for ethylene, different studies support a functional link between GSH and JA as well. Mutant *A. thaliana* plants without functional GR1 displayed a constitutive increase in oxidized glutathione disulfide (GSSG), which affected the expression of genes involved in JA metabolism dependent on day length conditions (Mhamdi et al., 2010). Expression levels of two JA signaling marker genes [plant defensin 1.2 (*PDF1.2*) and vegetative storage protein 2 (*VSP2*)] were significantly lower in GSH deficient *cad2-1* or *regulator of APX2 1-1* (*rax1-1*) mutants. Similar results were obtained when GSH biosynthesis was chemically inhibited by buthionine sulfoximine (BSO) in WT plants. In addition, microarray analysis revealed a multitude of genes involved in JA synthesis, activation and signaling to be differentially expressed in *cad2-1* mutants, indicating that the basal expression of JA-associated genes is affected by the content of GSH (Han et al., 2013). In the conditional oxidative stress signaling mutant *cat2* with H_2_O_2_-induced accumulation of GSSG under ambient air and moderate light conditions, the JA pathway was upregulated. However, this response was attenuated in a *cat2 cad2* double mutant, showing that GSH also regulates oxidative stress-induced JA-related gene expression in *A. thaliana* (Han et al., 2013). In addition to JA, SA was also shown to interact with GSH, as transgenic tobacco plants with an enhanced GSH content showed induction of SA-related genes such as *PATHOGENESIS-RELATED PROTEIN 1a* (*PR1a*; Ghanta et al., 2014). As ethylene and GSH are clearly intertwined during metal stress responses, it might be worthwhile to investigate the involvement of JA and SA in this interaction. In addition, JA signaling also involves the MAPK cascade MKK3/MPK6 (Takahashi et al., 2007) and NO^•^ does not only affect ethylene but also JA and SA signaling cascades (Mur et al., 2013). Therefore, it is recommended to further dissect the hormonal crosstalk affecting plant responses to metal stress (also reviewed by Thao et al., 2015) and address their interaction with oxidative stress and particularly GSH, the MAPK cascade and NO^•^ in future experiments.

## Concluding remarks

Ethylene is involved in many processes throughout the entire life cycle of plants, including responses to environmental stimuli such as metal exposure. Our current knowledge on the role of ethylene in metal-induced stress responses, as well as its integration within a broad network of signaling compounds, is gradually expanding. Recent evidence points toward an intimate link between ethylene, the cellular redox balance with GSH as important antioxidant and other phytohormones such as JA and SA, finally affecting plant metal sensitivity vs. tolerance. Nevertheless, much work remains to be done before this information can be applied in practice.

## Author contributions

EK, KS, JV and AC participated in the conception of the topic. EK and AC wrote the manuscript. All authors read and approved the final manuscript after critically revising it for important intellectual content.

## Funding

This work was supported by the Research Foundation Flanders (FWO) by a grant for EK and project [G0D3414]. Additional funding came from Hasselt University through a PhD grant for KS and the Methusalem project [08M03VGRJ].

### Conflict of interest statement

The authors declare that the research was conducted in the absence of any commercial or financial relationships that could be construed as a potential conflict of interest.

## References

[B1] Abeles F. B. Morgan P. W. Saltveit M. E. (1992). Ethylene in Plant Biology. San Diego, CA: Academic Press Inc.

[B2] Adams E. Turner J. (2010). COI1, a jasmonate receptor, is involved in ethylene-induced inhibition of *Arabidopsis* root growth in the light. J. Exp. Bot. 61, 4373–4386. 10.1093/jxb/erq240 20699268 PMC2955748

[B3] Alba R. Payton P. Fei Z. McQuinn R. Debbie P. Martin G. B.. (2005). Transcriptome and selected metabolite analyses reveal multiple points of ethylene control during tomato fruit development. Plant Cell 17, 2954–2965. 10.1105/tpc.105.036053 16243903 PMC1276022

[B4] Alloway B. J. (2012). Sources of heavy metals and metalloids in soils, in Heavy Metals in Soils: Trace Metals And Metalloids in Soils and Their Bioavailability, ed Alloway B. J. (Heidelberg: Springer), 11–50.

[B5] Alonso J. M. Hirayama T. Roman G. Nourizadeh S. Ecker J. R. (1999). EIN2, a bifunctional transducer of ethylene and stress responses in *Arabidopsis*. Science 284, 2148–2152. 10.1126/science.284.5423.2148 10381874

[B6] Anjum N. A. Hasanuzzaman M. Hossain M. A. Thangavel P. Roychoudhury A. Gill S. S.. (2015). Jacks of metal/metalloid chelation trade in plants – an overview. Front. Plant Sci. 6:192. 10.3389/fpls.2015.00192 25883598 PMC4382971

[B7] Arasimowicz-Jelonek M. Floryszak-Wieczorek J. Gwóźdź E. A. (2011). The message of nitric oxide in cadmium challenged plants. Plant Sci. 181, 612–620. 10.1016/j.plantsci.2011.03.019 21893258

[B8] Argueso C. T. Hansen M. Kieber J. J. (2007). Regulation of ethylene biosynthesis. J. Plant Growth Regul. 26, 92–105. 10.1007/s00344-007-0013-5

[B9] Arshad M. Saleem M. Hussain S. (2007). Perspectives of bacterial ACC deaminase in phytoremediation. Trends Biotechnol. 25, 356–362. 10.1016/j.tibtech.2007.05.005 17573137

[B10] Arteca J. M. Arteca R. N. (1999). A multi-responsive gene encoding 1-aminocyclopropane-1-carboxylate synthase (ACS6) in mature *Arabidopsis* leaves. Plant Mol. Biol. 39, 209–219. 10.1023/A:1006177902093 10080689

[B11] Arteca R. N. Arteca J. M. (2007). Heavy-metal-induced ethylene production in *Arabidopsis thaliana*. J. Plant Physiol. 164, 1480–1488. 10.1016/j.jplph.2006.09.006 17215058

[B12] Asgher M. Khan M. I. R. Anjum N. A. Khan N. A. (2015). Minimising toxicity of cadmium in plants - role of plant growth regulators. Protoplasma 252, 399–413. 10.1007/s00709-014-0710-4 25303855

[B13] Asgher M. Khan N. A. Khan M. I. R. Fatma M. Masood A. (2014). Ethylene production is associated with alleviation of cadmium-induced oxidative stress by sulfur in mustard types differing in ethylene sensitivity. Ecotoxicol. Environ. Saf. 106, 54–61. 10.1016/j.ecoenv.2014.04.017 24836878

[B14] Bartoli C. G. Casalongué C. A. Simontacchi M. Marquez-Garcia B. Foyer C. H. (2013). Interactions between hormone and redox signalling pathways in the control of growth and cross tolerance to stress. Environ. Exp. Bot. 94, 73–88. 10.1016/j.envexpbot.2012.05.003

[B15] Baxter A. Mittler R. Suzuki N. (2014). ROS as key players in plant stress signalling. J. Exp. Bot. 65, 1229–1240. 10.1093/jxb/ert375 24253197

[B16] Bisson M. M. A. Bleckmann A. Allekotte S. Groth G. (2009). EIN2, the central regulator of ethylene signalling, is localized at the ER membrane where it interacts with the ethylene receptor ETR1. Biochem. J. 424, 1–6. 10.1042/BJ20091102 19769567

[B17] Bisson M. M. A. Groth G. (2010). New insight in ethylene signaling: autokinase activity of ETR1 modulates the interaction of receptors and EIN2. Mol. Plant 3, 882–889. 10.1093/mp/ssq036 20591837

[B18] Bleecker A. B. Kende H. (2000). Ethylene: a gaseous signal molecule in plants. Annu. Rev. Cell Dev. Biol. 16, 1–18. 10.1146/annurev.cellbio.16.1.1 11031228

[B19] Bueso E. Alejandro S. Carbonell P. Perez-Amador M. A. Fayos J. Bellés J. M.. (2007). The lithium tolerance of the Arabidopsis *cat2* mutant reveals a cross-talk between oxidative stress and ethylene. Plant J. 52, 1052–1065. 10.1111/j.1365-313X.2007.03305.x 17931347

[B20] Cao F. Chen F. Sun H. Zhang G. Chen Z.-H. Wu F. (2014). Genome-wide transcriptome and functional analysis of two contrasting genotypes reveals key genes for cadmium tolerance in barley. BMC Genomics 15:611. 10.1186/1471-2164-15-611 25038590 PMC4117959

[B21] Cao S. Chen Z. Liu G. Jiang L. Yuan H. Ren G.. (2009). The *Arabidopsis Ethylene-Insensitive 2* gene is required for lead resistance. Plant Physiol. Biochem. 47, 308–312. 10.1016/j.plaphy.2008.12.013 19153049

[B22] Cao S. Jiang S. Zhang R. (2006). Evidence for a role of *Ethylene-Insensitive 2* gene in the regulation of the oxidative stress response in *Arabidopsis*. Acta Physiol. Plant. 28, 417–425. 10.1007/BF02706624

[B23] Carrió-Seguí A. Garcia-Molina A. Sanz A. Peñarrubia L. (2015). Defective copper transport in the *copt5* mutant affects cadmium tolerance. Plant Cell Physiol. 56, 442–454. 10.1093/pcp/pcu180 25432970

[B24] Cela J. Falk J. Munné-Bosch S. (2009). Ethylene signaling may be involved in the regulation of tocopherol biosynthesis in *Arabidopsis thaliana*. FEBS Lett. 583, 992–996. 10.1016/j.febslet.2009.02.036 19258016

[B25] Chae H. S. Kieber J. J. (2005). Eto brute? Role of ACS turnover in regulating ethylene biosynthesis. Trends Plant Sci. 10, 291–296. 10.1016/j.tplants.2005.04.006 15949763

[B26] Chae H. S. Lee W. S. (2001). Ethylene- and enzyme-mediated superoxide production and cell death in carrot cells grown under carbon starvation. Plant Cell Rep. 20, 256–261. 10.1007/s002990000307

[B27] Chen H.-J. Huang C.-S. Huang G.-J. Chow T.-J. Lin Y.-H. (2013). NADPH oxidase inhibitor diphenyleneiodonium and reduced glutathione mitigate ethephon-mediated leaf senescence, H_2_O_2_elevation and senescence-associated gene expression in sweet potato (*Ipomoea batatas*). J. Plant Physiol. 170, 1471–1483. 10.1016/j.jplph.2013.05.015 23834930

[B28] Chen Y.-A. Chi W.-C. Trinh N. N. Huang L.-Y. Chen Y.-C. Cheng K.-T.. (2014). Transcriptome profiling and physiological studies reveal a major role for aromatic amino acids in mercury stress tolerance in rice seedlings. PLoS ONE 9:e95163. 10.1371/journal.pone.0095163 24840062 PMC4026224

[B29] Chmielowska-Bąk J. Gzyl J. Ruciñska-Sobkowiak R. Arasimowicz-Jelonek M. Deckert J. (2014). The new insights into cadmium sensing. Front. Plant Sci. 5:245. 10.3389/fpls.2014.00245 24917871 PMC4042028

[B30] Chmielowska-Bąk J. Lefèvre I. Lutts S. Deckert J. (2013). Short term signaling responses in roots of young soybean seedlings exposed to cadmium stress. J. Plant Physiol. 170, 1585–1594. 10.1016/j.jplph.2013.06.019 23942356

[B31] Collin V. C. Eymery F. Genty B. Rey P. Havaux M. (2008). Vitamin E is essential for the tolerance of *Arabidopsis thaliana* to metal-induced oxidative stress. Plant Cell Environ. 31, 244–257. 10.1111/j.1365-3040.2007.01755.x 17996014

[B32] Corpas F. J. Leterrier M. Valderrama R. Airaki M. Chaki M. Palma J. M.. (2011). Nitric oxide imbalance provokes a nitrosative response in plants under abiotic stress. Plant Sci. 181, 604–611. 10.1016/j.plantsci.2011.04.005 21893257

[B33] Cuypers A. Smeets K. Vangronsveld J. (2009). Heavy metal stress in plants in Plant Stress Biology. From Genomics to Systems Biology, ed Hirt H. (Weinheim: Wiley-VCH Verlagsgesellschaft GmbH & Co. KGaA), 161–178.

[B34] DalCorso G. Manara A. Furini A. (2013). An overview of heavy metal challenge in plants: from roots to shoots. Metallomics 5, 1117–1132. 10.1039/c3mt00038a 23739766

[B35] Dat J. Vandenabeele S. Vranová E. Van Montagu M. Inzé D. Van Breusegem F. (2000). Dual action of the active oxygen species during plant stress responses. Cell. Mol. Life Sci. 57, 779–795. 10.1007/s000180050041 10892343 PMC11147059

[B36] de Jong A. Yakimova E. Kapchina V. M. Woltering E. J. (2002). A critical role for ethylene in hydrogen peroxide release during programmed cell death in tomato suspension cells. Planta 214, 537–545. 10.1007/s004250100654 11925037

[B37] De Martinis D. Koyama T. Chang K. (2015). Ethylene is all around. Front. Plant Sci. 6:76. 10.3389/fpls.2015.00076 25729386 PMC4325880

[B38] De Paepe A. Van Der Straeten D. (2005). Ethylene biosynthesis and signaling: an overview. Vitam. Horm. 72, 399–430. 10.1016/S0083-6729(05)72011-2 16492477

[B39] De Paepe A. Vuylsteke M. Van Hummelen P. Zabeau M. Van Der Straeten D. (2004). Transcriptional profiling by cDNA-AFLP and microarray analysis reveals novel insights into the early response to ethylene in *Arabidopsis*. Plant J. 39, 537–559. 10.1111/j.1365-313X.2004.02156.x 15272873

[B40] De Smet S. Cuypers A. Vangronsveld J. Remans T. (2015). Gene networks involved in hormonal control of root development in *Arabidopsis thaliana*: a framework for studying its disturbance by metal stress. Int. J. Mol. Sci. 16, 19195–19224. 10.3390/ijms160819195 26287175 PMC4581294

[B41] Diaz-Vivancos P. Barba-Espín G. Hernández J. A. (2013). Elucidating hormonal/ROS networks during seed germination: insights and perspectives. Plant Cell Rep. 32, 1491–1502. 10.1007/s00299-013-1473-7 23812175

[B42] Dietz K.-J. Vogel M. O. Viehhauser A. (2010). AP2/EREBP transcription factors are part of gene regulatory networks and integrate metabolic, hormonal and environmental signals in stress acclimation and retrograde signalling. Protoplasma 245, 3–14. 10.1007/s00709-010-0142-8 20411284

[B43] Dilley D. R. Wang Z. Kadirjan-Kalbach D. K. Ververidis F. Beaudry R. Padmanabhan K. (2013). 1-Aminocyclopropane-1-carboxylic acid oxidase reaction mechanism and putative post-translational activities of the ACCO protein. AoB Plants 5, plt031. 10.1093/aobpla/plt031 24244837 PMC3828642

[B44] Dolferus R. (2014). To grow or not to grow: a stressful decision for plants. Plant Sci. 229, 247–261. 10.1016/j.plantsci.2014.10.002 25443851

[B45] Dorling S. J. Leung S. Anderson C. W. N. Albert N. W. McManus M. T. (2011). Changes in 1-aminocyclopropane-1-carboxlate (ACC) oxidase expression and enzyme activity in response to excess manganese in white clover (*Trifolium repens* L.). Plant Physiol. Biochem. 49, 1013–1019. 10.1016/j.plaphy.2011.04.007 21530288

[B46] Fu S.-F. Chen P.-Y. Nguyen Q. T. T. Huang L.-Y. Zeng G.-R. Huang T.-L.. (2014). Transcriptome profiling of genes and pathways associated with arsenic toxicity and tolerance in *Arabidopsis*. BMC Plant Biol. 14:94. 10.1186/1471-2229-14-94 24734953 PMC4021232

[B47] Fujita M. Fujita Y. Noutoshi Y. Takahashi F. Narusaka Y. Yamaguchi-Shinozaki K.. (2006). Crosstalk between abiotic and biotic stress responses: a current view from the points of convergence in the stress signaling networks. Curr. Opin. Plant Biol. 9, 436–442. 10.1016/j.pbi.2006.05.014 16759898

[B48] Gane R. (1934). Production of ethylene by some ripening fruits. Nature 134, 1008 10.1038/1341008a0

[B49] Gao Z. Chen Y.-F. Randlett M. D. Zhao X.-C. Findell J. L. Kieber J. J.. (2003). Localization of the Raf-like kinase CTR1 to the endoplasmic reticulum of *Arabidopsis* through participation in ethylene receptor signaling complexes. J. Biol. Chem. 278, 34725–34732. 10.1074/jbc.M305548200 12821658

[B50] García M. J. Lucena C. Romera F. J. Alcántara E. Pérez-Vicente R. (2010). Ethylene and nitric oxide involvement in the up-regulation of key genes related to iron acquisition and homeostasis in Arabidopsis. J. Exp. Bot. 61, 3885–3899. 10.1093/jxb/erq203 20627899

[B51] Gergoff G. Chaves A. Bartoli C. G. (2010). Ethylene regulates ascorbic acid content during dark-induced leaf senescence. Plant Sci. 178, 207–212. 10.1016/j.plantsci.2009.12.003

[B52] Ghanta S. Datta R. Bhattacharyya D. Sinha R. Kumar D. Hazra S.. (2014). Multistep involvement of glutathione with salicylic acid and ethylene to combat environmental stress. J. Plant Physiol. 171, 940–950. 10.1016/j.jplph.2014.03.002 24913051

[B53] Glick B. R. (2014). Bacteria with ACC deaminase can promote plant growth and help to feed the world. Microbiol. Res. 169, 30–39. 10.1016/j.micres.2013.09.009 24095256

[B54] Glick B. R. Cheng Z. Czarny J. Duan J. (2007). Promotion of plant growth by ACC deaminase-producing soil bacteria. Eur. J. Plant Pathol. 119, 329–339. 10.1007/s10658-007-9162-4

[B55] Gratão P. L. Monteiro C. C. Carvalho R. F. Tezotto T. Piotto F. A. Peres L. E. P.. (2012). Biochemical dissection of *diageotropica* and *Never ripe* tomato mutants to Cd-stressful conditions. Plant Physiol. Biochem. 56, 79–96. 10.1016/j.plaphy.2012.04.009 22609458

[B56] Groppa M. D. Benavides M. P. Tomaro M. L. (2003). Polyamine metabolism in sunflower and wheat leaf discs under cadmium or copper stress. Plant Sci. 164, 293–299. 10.1016/S0168-9452(02)00412-026799504

[B57] Guan C. Ji J. Wu D. Li X. Jin C. Guan W. (2015). The glutathione synthesis may be regulated by cadmium-induced endogenous ethylene in *Lycium chinense*, and overexpression of an ethylene responsive transcription factor gene enhances tolerance to cadmium stress in tobacco. Mol. Breed. 35, 123 10.1007/s11032-015-0313-6

[B58] Guo H. Ecker J. R. (2003). Plant responses to ethylene gas are mediated by SCF^EBF1∕EBF2^-dependent proteolysis of EIN3 transcription factor. Cell 115, 667–677. 10.1016/S0092-8674(03)00969-3 14675532

[B59] Hahn A. Harter K. (2009). Mitogen-activated protein kinase cascades and ethylene: signaling, biosynthesis, or both? Plant Physiol. 149, 1207–1210. 10.1104/pp.108.132241 19109412 PMC2649397

[B60] Han L. Li G.-J. Yang K.-Y. Mao G. Wang R. Liu Y.. (2010). Mitogen-activated protein kinase 3 and 6 regulate *Botrytis cinerea*-induced ethylene production in *Arabidopsis*. Plant J. 64, 114–127. 10.1111/j.1365-313x.2010.04318.x 20659280

[B61] Han Y. Mhamdi A. Chaouch S. Noctor G. (2013). Regulation of basal and oxidative stress-triggered jasmonic acid-related gene expression by glutathione. Plant Cell Environ. 36, 1135–1146. 10.1111/pce.12048 23210597

[B62] Hänsch R. Mendel R. R. (2009). Physiological functions of mineral micronutrients (Cu, Zn, Mn, Fe, Ni, Mo, B, Cl). Curr. Opin. Plant Biol. 12, 259–266. 10.1016/j.pbi.2009.05.006 19524482

[B63] Herbette S. Taconnat L. Hugouvieux V. Piette L. Magniette M. -L. M. Cuine S.. (2006). Genome-wide transcriptome profiling of the early cadmium response of *Arabidopsis* roots and shoots. Biochimie 88, 1751–1765. 10.1016/j.biochi.2006.04.018 16797112

[B64] Hernández L. E. Sobrino-Plata J. Montero-Palmero M. B. Carrasco-Gil S. Flores-Cáceres M. L. Ortega-Villasante C.. (2015). Contribution of glutathione to the control of cellular redox homeostasis under toxic metal and metalloid stress. J. Exp. Bot. 66, 2901–2911. 10.1093/jxb/erv063 25750419

[B65] Hirayama T. Kieber J. J. Hirayama N. Kogan M. Guzman P. Nourizadeh S.. (1999). RESPONSIVE-TO-ANTAGONIST1, a Menkes/Wilson disease–related copper transporter, is required for ethylene signaling in *Arabidopsis*. Cell 97, 383–393. 10.1016/S0092-8674(00)80747-3 10319818

[B66] Hua J. Meyerowitz E. M. (1998). Ethylene responses are negatively regulated by a receptor gene family in *Arabidopsis thaliana*. Cell 94, 261–271. 10.1016/S0092-8674(00)81425-7 9695954

[B67] Iakimova E. T. Woltering E. J. Kapchina-Toteva V. M. Harren F. J. M. Cristescu S. M. (2008). Cadmium toxicity in cultured tomato cells - Role of ethylene, proteases and oxidative stress in cell death signaling. Cell Biol. Int. 32, 1521–1529. 10.1016/j.cellbi.2008.08.021 18801448

[B68] Iqbal N. Khan N. A. Nazar R. Teixeira da Silva J. A. (2012). Ethylene-stimulated photosynthesis results from increased nitrogen and sulfur assimilation in mustard types that differ in photosynthetic capacity. Environ. Exp. Bot. 78, 84–90. 10.1016/j.envexpbot.2011.12.025

[B69] Iqbal N. Masood A. Khan M. I. R. Asgher M. Fatma M. Khan N. A. (2013b). Cross-talk between sulfur assimilation and ethylene signaling in plants. Plant Signal. Behav. 8, 104–112. 10.4161/psb.22478 23104111 PMC3745555

[B70] Iqbal N. Trivellini A. Masood A. Ferrante A. Khan N. A. (2013a). Current understanding on ethylene signaling in plants: the influence of nutrient availability. Plant Physiol. Biochem. 73, 128–138. 10.1016/j.plaphy.2013.09.011 24095919

[B71] Jakubowicz M. Gałañska H. Nowak W. Sadowski J. (2010). Exogenously induced expression of ethylene biosynthesis, ethylene perception, phospholipase D, and Rboh-oxidase genes in broccoli seedlings. J. Exp. Bot. 61, 3475–3491. 10.1093/jxb/erq177 20581125 PMC2905205

[B72] Järup L. (2003). Hazards of heavy metal contamination. Br. Med. Bull. 68, 167–182. 10.1093/bmb/ldg032 14757716

[B73] Joo S. Liu Y. Lueth A. Zhang S. (2008). MAPK phosphorylation-induced stabilization of ACS6 protein is mediated by the non-catalytic C-terminal domain, which also contains the *cis*-determinant for rapid degradation by the 26S proteasome pathway. Plant J. 54, 129–140. 10.1111/j.1365-313X.2008.03404.x 18182027

[B74] Jozefczak M. Remans T. Vangronsveld J. Cuypers A. (2012). Glutathione is a key player in metal-induced oxidative stress defenses. Int. J. Mol. Sci. 13, 3145–3175. 10.3390/ijms13033145 22489146 PMC3317707

[B75] Ju C. Chang C. (2012). Advances in ethylene signalling: protein complexes at the endoplasmic reticulum membrane. AoB Plants 2012:pls031. 10.1093/aobpla/pls031 23119138 PMC3485614

[B76] Ju C. Yoon G. M. Shemansky J. M. Lin D. Y. Ying I. Chang J.. (2012). CTR1 phosphorylates the central regulator EIN2 to control ethylene hormone signaling from the ER membrane to the nucleus in *Arabidopsis*. Proc. Natl. Acad. Sci. U.S.A. 109, 19486–19491. 10.1073/pnas.1214848109 23132950 PMC3511113

[B77] Kacperska A. (2004). Sensor types in signal transduction pathways in plant cells responding to abiotic stressors: do they depend on stress intensity? Physiol. Plant. 122, 159–168. 10.1111/j.0031-9317.2004.00388.x

[B78] Keunen E. Schellingen K. Van Der Straeten D. Remans T. Colpaert J. Vangronsveld J.. (2015). ALTERNATIVE OXIDASE1a modulates the oxidative challenge during moderate Cd exposure in *Arabidopsis thaliana* leaves. J. Exp. Bot. 66, 2967–2977. 10.1093/jxb/erv035 25743159

[B79] Khan M. I. R. Khan N. A. (2014). Ethylene reverses photosynthetic inhibition by nickel and zinc in mustard through changes in PS II activity, photosynthetic nitrogen use efficiency, and antioxidant metabolism. Protoplasma 251, 1007–1019. 10.1007/s00709-014-0610-7 24477804

[B80] Khan M. I. R. Nazir F. Asgher M. Per T. S. Khan N. A. (2015). Selenium and sulfur influence ethylene formation and alleviate cadmium-induced oxidative stress by improving proline and glutathione production in wheat. J. Plant Physiol. 173, 9–18. 10.1016/j.jplph.2014.09.011 25462073

[B81] Kieber J. J. Rothenberg M. Roman G. Feldmann K. A. Ecker J. R. (1993). *CTR1*, a negative regulator of the ethylene response pathway in Arabidopsis, encodes a member of the Raf family of protein kinases. Cell 72, 427–441. 10.1016/0092-8674(93)90119-B 8431946

[B82] Kim J. Patterson S. E. Binder B. M. (2013). Reducing jasmonic acid levels causes *ein2* mutants to become ethylene responsive. FEBS Lett. 587, 226–230. 10.1016/j.febslet.2012.11.030 23219920

[B83] Kim Y. S. Choi D. Lee M. M. Lee S. H. Kim W. T. (1998). Biotic and abiotic stress-related expression of 1-aminocyclopropane-1-carboxylate oxidase gene family in *Nicotiana glutinosa* L. Plant Cell Physiol. 39, 565–573. 10.1093/oxfordjournals.pcp.a029406 9697341

[B84] Kohli A. Sreenivasulu N. Lakshmanan P. Kumar P. P. (2013). The phytohormone crosstalk paradigm takes center stage in understanding how plants respond to abiotic stresses. Plant Cell Rep. 32, 945–957. 10.1007/s00299-013-1461-y 23749097

[B85] Lei X. -Y. Zhu R. -Y. Zhang G. -Y. Dai Y -R. (2003). Possible involvement of the mitochondrial alternative pathway in ethylene-induced apoptosis in tomato protoplasts. Plant Growth Regul. 41, 111–116. 10.1023/A:1027355502538

[B86] Lequeux H. Hermans C. Lutts S. Verbruggen N. (2010). Response to copper excess in *Arabidopsis thaliana*: impact on the root system architecture, hormone distribution, lignin accumulation and mineral profile. Plant Physiol. Biochem. 48, 673–682. 10.1016/j.plaphy.2010.05.005 20542443

[B87] Li G. Meng X. Wang R. Mao G. Han L. Liu Y.. (2012). Dual-level regulation of ACC synthase activity by MPK3/MPK6 cascade and its downstream WRKY transcription factor during ethylene induction in Arabidopsis. PLoS Genet. 8:e1002767. 10.1371/journal.pgen.1002767 22761583 PMC3386168

[B88] Liang X. Shen N. F. Theologis A. (1996). Li^+^-regulated 1-aminocyclopropane-1-carboxylate synthase gene expression in *Arabidopsis thaliana*. Plant J. 10, 1027–1036. 10.1046/j.1365-313X.1996.10061027.x 9011084

[B89] Lin Z. Zhong S. Grierson D. (2009). Recent advances in ethylene research. J. Exp. Bot. 60, 3311–3336. 10.1093/jxb/erp204 19567479

[B90] Liu X. M. Kim K. E. Kim K. C. Nguyen X. C. Han H. J. Jung M. S.. (2010). Cadmium activates Arabidopsis MPK3 and MPK6 via accumulation of reactive oxygen species. Phytochemistry 71, 614–618. 10.1016/j.phytochem.2010.01.005 20116811

[B91] Liu Y. Zhang S. (2004). Phosphorylation of 1-aminocyclopropane-1-carboxylic acid synthase by MPK6, a stress-responsive mitogen-activated protein kinase, induces ethylene biosynthesis in Arabidopsis. Plant Cell 16, 3386–3399. 10.1105/tpc.104.026609 15539472 PMC535880

[B92] Lopes M. S. Iglesia-Turiño S. Cabrera-Bosquet L. Serret M. D. Bort J. Febrero A.. (2013). Molecular and physiological mechanisms associated with root exposure to mercury in barley. Metallomics 5, 1305–1315. 10.1039/c3mt00084b 23925371

[B93] Lu W. P. Kirkham M. B. (1991). Genotypic tolerance to metals as indicated by ethylene production. Water Air Soil Pollut. 57–58, 605–615. 10.1007/BF00282924

[B94] Lynch J. Brown K. M. (1997). Ethylene and plant responses to nutritional stress. Physiol. Plant. 100, 613–619. 10.1111/j.1399-3054.1997.tb03067.x

[B95] Maksymiec W. (2011). Effects of jasmonate and some other signalling factors on bean and onion growth during the initial phase of cadmium action. Biol. Plant. 55, 112–118. 10.1007/s10535-011-0015-9

[B96] Maksymiec W. Krupa Z. (2006). The effects of short-term exposition to Cd, excess Cu ions and jasmonate on oxidative stress appearing in Arabidopsis thaliana. Environ. Exp. Bot. 57, 187–194. 10.1016/j.envexpbot.2005.05.006

[B97] Maksymiec W. Wianowska D. Dawidowicz A. L. Radkiewicz S. Mardarowicz M. Krupa Z. (2005). The level of jasmonic acid in *Arabidopsis thaliana* and *Phaseolus coccineus* plants under heavy metal stress. J. Plant Physiol. 162, 1338–1346. 10.1016/j.jplph.2005.01.013 16425452

[B98] Masood A. Iqbal N. Khan N. A. (2012). Role of ethylene in alleviation of cadmium-induced photosynthetic capacity inhibition by sulphur in mustard. Plant Cell Environ. 35, 524–533. 10.1111/j.1365-3040.2011.02432.x 21950968

[B99] Mazorra Morales L. M. Senn M. E. Grozeff G. E. G. Fanello D. D. Carrión C. A. Núñez M.. (2014). Impact of brassinosteroids and ethylene on ascorbic acid accumulation in tomato leaves. Plant Physiol. Biochem. 74, 315–322. 10.1016/j.plaphy.2013.11.021 24342083

[B100] McDonnell L. Plett J. M. Andersson-Gunnerås S. Kozela C. Dugardeyn J. Van Der Straeten D.. (2009). Ethylene levels are regulated by a plant encoded 1-aminocyclopropane-1-carboxylic acid deaminase. Physiol. Plant. 136, 94–109. 10.1111/j.1399-3054.2009.01208.x 19508369

[B101] Merchante C. Alonso J. M. Stepanova A. N. (2013). Ethylene signaling: simple ligand, complex regulation. Curr. Opin. Plant Biol. 16, 554–560. 10.1016/j.pbi.2013.08.001 24012247

[B102] Mersmann S. Bourdais G. Rietz S. Robatzek S. (2010). Ethylene signaling regulates accumulation of the FLS2 receptor and is required for the oxidative burst contributing to plant immunity. Plant Physiol. 154, 391–400. 10.1104/pp.110.154567 20592040 PMC2938167

[B103] Mertens J. Vangronsveld J. Van Der Straeten D. Van Poucke M. (1999). Effects of copper and zinc on the ethylene production of *Arabidopsis thaliana*, in Biology and Biotechnology of the Plant Hormone Ethylene II, eds Kanellis A. K. Chang C. Klee H. Bleecker A. B. Pech J. C. Grierson D. (Dordrecht: Kluwer Academic Publishers), 333–338.

[B104] Mhamdi A. Hager J. Chaouch S. Queval G. Han Y. Taconnat L.. (2010). Arabidopsis GLUTATHIONE REDUCTASE1 plays a crucial role in leaf responses to intracellular hydrogen peroxide and in ensuring appropriate gene expression through both salicylic acid and jasmonic acid signaling pathways. Plant Physiol. 153, 1144–1160. 10.1104/pp.110.153767 20488891 PMC2899936

[B105] Mittler R. (2006). Abiotic stress, the field environment and stress combination. Trends Plant Sci. 11, 15–19. 10.1016/j.tplants.2005.11.002 16359910

[B106] Mittler R. Vanderauwera S. Gollery M. Van Breusegem F. (2004). Reactive oxygen gene network of plants. Trends Plant Sci. 9, 490–498. 10.1016/j.tplants.2004.08.009 15465684

[B107] Mittler R. Vanderauwera S. Suzuki N. Miller G. Tognetti V. B. Vandepoele K.. (2011). ROS signaling: the new wave? Trends Plant Sci. 16, 300–309. 10.1016/j.tplants.2011.03.007 21482172

[B108] Møller I. M. Jensen P. E. Hansson A. (2007). Oxidative modifications to cellular components in plants. Annu. Rev. Plant Biol. 58, 459–481. 10.1146/annurev.arplant.58.032806.103946 17288534

[B109] Montero-Palmero M. B. Martín-Barranco A. Escobar C. Hernández L. E. (2014a). Early transcriptional responses to mercury: a role for ethylene in mercury-induced stress. New Phytol. 201, 116–130. 10.1111/nph.12486 24033367

[B110] Montero-Palmero M. B. Ortega-Villasante C. Escobar C. Hernández L. E. (2014b). Are plant endogenous factors like ethylene modulators of the early oxidative stress induced by mercury? Front. Environ. Sci. 2:34 10.3389/fenvs.2014.00034

[B111] Mur L. A. J. Prats E. Pierre S. Hall M. A. Hebelstrup K. H. (2013). Integrating nitric oxide into salicylic acid and jasmonic acid/ethylene plant defense pathways. Front. Plant Sci. 4:215. 10.3389/fpls.2013.00215 23818890 PMC3694216

[B112] Murphy L. J. Robertson K. N. Harroun S. G. Brosseau C. L. Werner-Zwanziger U. Moilanen J.. (2014). A simple complex on the verge of breakdown: Isolation of the elusive cyanoformate ion. Science 344, 75–78. 10.1126/science.1250808 24700853

[B113] Nagarajan V. K. Smith A. P. (2011). Ethylene's role in phosphate starvation signaling: more than just a root growth regulator. Plant Cell Physiol. 53, 277–286. 10.1093/pcp/pcr186 22199374

[B114] Naranjo M. A. Romero C. Bellés J. M. Montesinos C. Vicente O. Serrano R. (2003). Lithium treatment induces a hypersensitive-like response in tobacco. Planta 217, 417–424. 10.1007/s00425-003-1017-4 14520568

[B115] Neljubov D. (1901). Ueber die horizontale Nutation der Stengel von *Pisum sativum* und einiger anderen Pflanzen. Beiheifte Bot. Zentralblatt 10, 128–139.

[B116] Opdenakker K. Remans T. Vangronsveld J. Cuypers A. (2012). Mitogen-activated protein (MAP) kinases in plant metal stress: regulation and responses in comparison to other biotic and abiotic stresses. Int. J. Mol. Sci. 13, 7828–7853. 10.3390/ijms13067828 22837729 PMC3397561

[B117] Ouaked F. Rozhon W. Lecourieux D. Hirt H. (2003). A MAPK pathway mediates ethylene signaling in plants. EMBO J. 22, 1282–1288. 10.1093/emboj/cdg131 12628921 PMC151067

[B118] Overmyer K. Brosché M. Kangasjärvi J. (2003). Reactive oxygen species and hormonal control of cell death. Trends Plant Sci. 8, 335–342. 10.1016/S1360-1385(03)00135-3 12878018

[B119] Peng H.-P. Lin T.-Y. Wang N.-N. Shih M.-C. (2005). Differential expression of genes encoding 1-aminocyclopropane-1-carboxylate synthase in *Arabidopsis* during hypoxia. Plant Mol. Biol. 58, 15–25. 10.1007/s11103-005-3573-4 16028113

[B120] Petrov V. D. Van Breusegem F. (2012). Hydrogen peroxide - a central hub for information flow in plant cells. AoB Plants 2012, pls014. 10.1093/aobpla/pls014 22708052 PMC3366437

[B121] Pierik R. Tholen D. Poorter H. Visser E. J. W. Voesenek L. A. C. J. (2006). The Janus face of ethylene: growth inhibition and stimulation. Trends Plant Sci. 11, 176–183. 10.1016/j.tplants.2006.02.006 16531097

[B122] Qiao H. Chang K. N. Yazaki J. Ecker J. R. (2009). Interplay between ethylene, ETP1/ETP2 F-box proteins, and degradation of EIN2 triggers ethylene responses in *Arabidopsis*. Genes Dev. 23, 512–521. 10.1101/gad.1765709 19196655 PMC2648649

[B123] Remans T. Opdenakker K. Smeets K. Mathijsen D. Vangronsveld J. Cuypers A. (2010). Metal-specific and NADPH oxidase dependent changes in lipoxygenase and NADPH oxidase gene expression in *Arabidopsis thaliana* exposed to cadmium or excess copper. Funct. Plant Biol. 37, 532–544. 10.1071/FP0919426475193

[B124] Remans T. Thijs S. Truyens S. Weyens N. Schellingen K. Keunen E.. (2012). Understanding the development of roots exposed to contaminants and the potential of plant-associated bacteria for optimization of growth. Ann. Bot. 110, 239–252. 10.1093/aob/mcs105 22634257 PMC3394651

[B125] Resnick J. S. Rivarola M. Chang C. (2008). Involvement of RTE1 in conformational changes promoting ETR1 ethylene receptor signaling in *Arabidopsis*. Plant J. 56, 423–431. 10.1111/j.1365-313X.2008.03615.x 18643990 PMC2575083

[B126] Resnick J. S. Wen C. -K. Shockey J. A. Chang C. (2006). REVERSION-TO-ETHYLENE SENSITIVITY1, a conserved gene that regulates ethylene receptor function in *Arabidopsis*. Proc. Natl. Acad. Sci. U.S.A. 103, 7917–7922. 10.1073/pnas.0602239103 16682642 PMC1458508

[B127] Rodríguez-Serrano M. Romero-Puertas M. C. Pazmiño D. M. Testillano P. S. Risueño M. C. del Río L. A.. (2009). Cellular response of pea plants to cadmium toxicity: Cross talk between reactive oxygen species, nitric oxide, and calcium. Plant Physiol. 150, 229–243. 10.1104/pp.108.131524 19279198 PMC2675729

[B128] Rodríguez-Serrano M. Romera-Puertas M. C. Zabalza A. Corpas F. J. Gómez M. Del Río L. A.. (2006). Cadmium effect on oxidative metabolism of pea (*Pisum sativum* L.) roots. Imaging of reactive oxygen species and nitric oxide accumulation *in vivo*. Plant Cell Environ. 29, 1532–1534. 16898016 10.1111/j.1365-3040.2006.01531.x

[B129] Ruduś I. Sasiak M. Kêpczyñski J. (2012). Regulation of ethylene biosynthesis at the level of 1-aminocyclopropane-1-carboxylate oxidase (ACO) gene. Acta Physiol. Plant. 35, 295–307. 10.1007/s11738-012-1096-6

[B130] Schellingen K. Van Der Straeten D. Remans T. Loix C. Vangronsveld J. Cuypers A. (2015a). Ethylene biosynthesis is involved in the early oxidative challenge induced by moderate Cd exposure in *Arabidopsis thaliana*. Environ. Exp. Bot. 117, 1–11. 10.1016/j.envexpbot.2015.04.005 25743159

[B131] Schellingen K. Van Der Straeten D. Remans T. Vangronsveld J. Keunen E. Cuypers A. (2015b). Ethylene signalling is mediating the early cadmium-induced oxidative challenge in *Arabidopsis thaliana*. Plant Sci. 239, 137–146. 10.1016/j.plantsci.2015.07.015 26398798

[B132] Schellingen K. Van Der Straeten D. Vandenbussche F. Prinsen E. Remans T. Vangronsveld J.. (2014). Cadmium-induced ethylene production and responses in *Arabidopsis thaliana* rely on *ACS2* and *ACS6* gene expression. BMC Plant Biol. 14:214. 10.1186/s12870-014-0214-6 25082369 PMC4236733

[B133] Schnaubelt D. Queval G. Dong Y. Diaz-Vivancos P. Makgopa M. E. Howell G.. (2015). Low glutathione regulates gene expression and the redox potentials of the nucleus and cytosol in *Arabidopsis thaliana*. Plant Cell Environ. 38, 266–279. 10.1111/pce.12252 24329757

[B134] Schützendübel A. Polle A. (2002). Plant responses to abiotic stresses: heavy metal-induced oxidative stress and protection by mycorrhization. J. Exp. Bot. 53, 1351–1365. 10.1093/jexbot/53.372.1351 11997381

[B135] Seth C. S. Remans T. Keunen E. Jozefczak M. Gielen H. Opdenakker K.. (2012). Phytoextraction of toxic metals: a central role for glutathione. Plant Cell Environ. 35, 334–346. 10.1111/j.1365-3040.2011.02338.x 21486307

[B136] Shakeel S. N. Gao Z. Amir M. Chen Y.-F. Rai M. I. Haq N. U.. (2015). Ethylene regulates levels of ethylene receptor/CTR1 signaling complexes in *Arabidopsis thaliana*. J. Biol. Chem. 290, 12415–12424. 10.1074/jbc.M115.652503 25814663 PMC4424370

[B137] Sharma S. S. Dietz K.-J. (2009). The relationship between metal toxicity and cellular redox imbalance. Trends Plant Sci. 14, 43–50. 10.1016/j.tplants.2008.10.007 19070530

[B138] Skottke K. R. Yoon G. M. Kieber J. J. DeLong A. (2011). Protein phosphatase 2A controls ethylene biosynthesis by differentially regulating the turnover of ACC synthase isoforms. PLoS Genet. 7:e1001370. 10.1371/journal.pgen.1001370 21533019 PMC3080859

[B139] Song S. Qi T. Wasternack C. Xie D. (2014). Jasmonate signaling and crosstalk with gibberellin and ethylene. Curr. Opin. Plant Biol. 21, 112–119. 10.1016/j.pbi.2014.07.005 25064075

[B140] Srivastava A. K. Venkatachalam P. Raghothama K. G. Sahi S. V. (2007). Identification of lead-regulated genes by suppression subtractive hybridization in the heavy metal accumulator *Sesbania drummondii*. Planta 225, 1353–1365. 10.1007/s00425-006-0445-3 17143618

[B141] Steffens B. (2014). The role of ethylene and ROS in salinity, heavy metal, and flooding responses in rice. Front. Plant Sci. 5:685. 10.3389/fpls.2014.00685 25538719 PMC4255495

[B142] Sun P. Tian Q.-Y. Chen J. Zhang W.-H. (2010). Aluminium-induced inhibition of root elongation in *Arabidopsis* is mediated by ethylene and auxin. J. Exp. Bot. 61, 347–356. 10.1093/jxb/erp306 19858117 PMC2803203

[B143] Sun P. Tian Q.-Y. Zhao M.-G. Dai X.-Y. Huang J.-H. Li L.-H.. (2007). Aluminum-induced ethylene production is associated with inhibition of root elongation in *Lotus japonicus* L. Plant Cell Physiol. 48, 1229–1235. 10.1093/pcp/pcm077 17573361

[B144] Sun X. Guo L. (2013). Relationship between cadmium-induced root subapical hair development and ethylene biosynthesis in oilseed rape seedlings. Acta Biol. Cracoviensia Ser. Bot. 55, 68–75. 10.2478/abcsb-2013-0018

[B145] Suzuki N. Miller G. Morales J. Shulaev V. Torres M. A. Mittler R. (2011). Respiratory burst oxidases: the engines of ROS signaling. Curr. Opin. Plant Biol. 14, 691–699. 10.1016/j.pbi.2011.07.014 21862390

[B146] Takahashi F. Yoshida R. Ichimura K. Mizoguchi T. Seo S. Yonezawa M.. (2007). The mitogen-activated protein kinase cascade MKK3-MPK6 is an important part of the jasmonate signal transduction pathway in *Arabidopsis*. Plant Cell 19, 805–818. 10.1105/tpc.106.046581 17369371 PMC1867372

[B147] Thain S. C. Vandenbussche F. Laarhoven L. J. J. Dowson-Day M. J. Wang Z.-Y. Tobin E. M.. (2004). Circadian rhythms of ethylene emission in Arabidopsis. Plant Physiol. 136, 3751–3761. 10.1104/pp.104.042523 15516515 PMC527172

[B148] Thao N. P. Khan M. I. R. Binh N. Thu A. Lan X. Hoang T.. (2015). Role of ethylene and its cross talk with other signaling molecules in plant responses to heavy metal stress. Plant Cell Environ. 169, 73–84. 10.1104/pp.15.00663 26246451 PMC4577409

[B149] Tian Q. Zhang X. Ramesh S. Gilliham M. Tyerman S. D. Zhang W.-H. (2014). Ethylene negatively regulates aluminium-induced malate efflux from wheat roots and tobacco cells transformed with *TaALMT1*. J. Exp. Bot. 65, 2415–2426. 10.1093/jxb/eru123 24668874 PMC4036508

[B150] Trinh N.-N. Huang T.-L. Chi W.-C. Fu S.-F. Chen C.-C. Huang H.-J. (2014). Chromium stress response effect on signal transduction and expression of signaling genes in rice. Physiol. Plant. 150, 205–224. 10.1111/ppl.12088 24033343

[B151] Tsuchisaka A. Yu G. Jin H. Alonso J. M. Ecker J. R. Zhang X.. (2009). A combinatorial interplay among the 1-aminocyclopropane-1-carboxylate isoforms regulates ethylene biosynthesis in *Arabidopsis thaliana*. Genetics 183, 979–1003. 10.1534/genetics.109.107102 19752216 PMC2778992

[B152] Vahala J. Schlagnhaufer C. D. Pell E. J. (1998). Induction of an ACC synthase cDNA by ozone in light-grown *Arabidopsis thaliana* leaves. Physiol. Plant. 103, 45–50. 10.1034/j.1399-3054.1998.1030106.x

[B153] Vandenbussche F. Vaseva I. Vissenberg K. Van Der Straeten D. (2012). Ethylene in vegetative development: a tale with a riddle. New Phytol. 194, 895–909. 10.1111/j.1469-8137.2012.04100.x 22404712

[B154] Vandenbussche F. Vriezen W. H. Smalle J. Laarhoven L. J. J. Harren F. J. M. Van Der Straeten D. (2003). Ethylene and auxin control the Arabidopsis response to decreased light intensity. Plant Physiol. 133, 517–527. 10.1104/pp.103.022665 12972669 PMC219028

[B155] Van de Poel B. Bulens I. Hertog M. L. Nicolai B. M. Geeraerd A. H. (2014). A transcriptomics-based kinetic model for ethylene biosynthesis in tomato (*Solanum lycopersicum*) fruit: development, validation and exploration of novel regulatory mechanisms. New Phytol. 202, 952–963. 10.1111/nph.12685 24443955

[B156] Van de Poel B. Bulens I. Markoula A. Hertog M. L. Dreesen R. Wirtz M.. (2012). Targeted systems biology profiling of tomato fruit reveals coordination of the Yang cycle and a distinct regulation of ethylene biosynthesis during postclimacteric ripening. Plant Physiol. 160, 1498–1514. 10.1104/pp.112.206086 22977280 PMC3490579

[B157] Van de Poel B. Smet D. Van Der Straeten D. (2015). Ethylene and hormonal crosstalk in vegetative growth and development. Plant Physiol. 169, 61–72. 10.1104/pp.15.00724 26232489 PMC4577414

[B158] Van de Poel B. Van Der Straeten D. (2014). 1-aminocyclopropane-1-carboxylic acid (ACC) in plants: more than just the precursor of ethylene! Front. Plant Sci. 5:640. 10.3389/fpls.2014.00640 25426135 PMC4227472

[B159] Vangronsveld J. Herzig R. Weyens N. Boulet J. Adriaensen K. Ruttens A.. (2009). Phytoremediation of contaminated soils and groundwater: lessons from the field. Environ. Sci. Pollut. Res. Int. 16, 765–794. 10.1007/s11356-009-0213-6 19557448

[B160] Vassilev A. Lidon F. Scotti P. Da Graca M. Yordanov I. (2004). Cadmium-induced changes in chloroplast lipids and photosystem activities in barley plants. Biol. Plant. 48, 153–156. 10.1023/B:BIOP.0000024295.27419.89

[B161] Verbruggen N. Hermans C. Schat H. (2009). Mechanisms to cope with arsenic or cadmium excess in plants. Curr. Opin. Plant Biol. 12, 364–372. 10.1016/j.pbi.2009.05.001 19501016

[B162] Wang H. Liang X. Wan Q. Wang X. Bi Y. (2009). Ethylene and nitric oxide are involved in maintaining ion homeostasis in *Arabidopsis* callus under salt stress. Planta 230, 293–307. 10.1007/s00425-009-0946-y 19455351

[B163] Weber M. Trampczynska A. Clemens S. (2006). Comparative transcriptome analysis of toxic metal responses in *Arabidopsis thaliana* and the Cd^2+^-hypertolerant facultative metallophyte *Arabidopsis halleri*. Plant Cell Environ. 29, 950–963. 10.1111/j.1365-3040.2005.01479.x 17087478

[B164] Weyens N. van der Lelie D. Taghavi S. Newman L. Vangronsveld J. (2009). Exploiting plant-microbe partnerships to improve biomass production and remediation. Trends Biotechnol. 27, 591–598. 10.1016/j.tibtech.2009.07.006 19683353

[B165] Xu J. Li Y. Wang Y. Liu H. Lei L. Yang H.. (2008). Activation of MAPK kinase 9 induces ethylene and camalexin biosynthesis and enhances sensitivity to salt stress in *Arabidopsis*. J. Biol. Chem. 283, 26996–27006. 10.1074/jbc.M801392200 18693252

[B166] Xu S. S. Lin S. Z. Lai Z. X. (2015). Cadmium impairs iron homeostasis in *Arabidopsis thaliana* by increasing the polysaccharide contents and the iron-binding capacity of root cell walls. Plant Soil 392, 71–85. 10.1007/s11104-015-2443-3

[B167] Yakimova E. T. Kapchina-Toteva V. M. Woltering E. J. (2007). Signal transduction events in aluminum-induced cell death in tomato suspension cells. J. Plant Physiol. 164, 702–708. 10.1016/j.jplph.2006.03.018 16781012

[B168] Yamagami T. Tsuchisaka A. Yamada K. Haddon W. F. Harden L. A. Theologis A. (2003). Biochemical diversity among the 1-amino-cyclopropane-1-carboxylate synthase isozymes encoded by the *Arabidopsis* gene family. J. Biol. Chem. 278, 49102–49112. 10.1074/jbc.M308297200 12968022

[B169] Yamauchi M. Peng X. X. (1995). Iron toxicity and stress-induced ethylene production in rice leaves. Plant Soil 173, 21–28. 10.1007/BF00155514

[B170] Yang S. F. Hoffman N. E. (1984). Ethylene biosynthesis and its regulation in higher plants. Annu. Rev. Plant Physiol. 35, 155–189. 10.1146/annurev.pp.35.060184.001103

[B171] Yoo S.-D. Cho Y. Sheen J. (2009). Emerging connections in the ethylene signaling network. Trends Plant Sci. 14, 270–279. 10.1016/j.tplants.2009.02.007 19375376 PMC3063992

[B172] Yoo S.-D. Cho Y.-H. Tena G. Xiong Y. Sheen J. (2008). Dual control of nuclear EIN3 by bifurcate MAPK cascades in C_2_H_4_ signalling. Nature 451, 789–795. 10.1038/nature06543 18273012 PMC3488589

[B173] Yoon G. M. Kieber J. J. (2013). 1-Aminocyclopropane-1-carboxylic acid as a signalling molecule in plants. AoB Plants 5, plt017. 10.1093/aobpla/plt01724078672

[B174] Yoshida S. Tamaoki M. Ioki M. Ogawa D. Sato Y. Aono M.. (2009). Ethylene and salicylic acid control glutathione biosynthesis in ozone-exposed *Arabidopsis thaliana*. Physiol. Plant. 136, 284–298. 10.1111/j.1399-3054.2009.01220.x 19453511

[B175] Zhang Y. He Q. Zhao S. Huang L. Hao L. (2014). Arabidopsis *ein2-1* and *npr1-1* response to Al stress. Bull. Environ. Contam. Toxicol. 93, 78–83. 10.1007/s00128-014-1249-y 24619362

[B176] Zhao Q. Guo H.-W. (2011). Paradigms and paradox in the ethylene signaling pathway and interaction network. Mol. Plant 4, 626–634. 10.1093/mp/ssr042 21690206

[B177] Zhou Z. S. Yang S. N. Li H. Zhu C. C. Liu Z. P. Yang Z. M. (2013). Molecular dissection of mercury-responsive transcriptome and sense/antisense genes in *Medicago truncatula*. J. Hazard. Mater. 252–253, 123–131. 10.1016/j.jhazmat.2013.02.011 23500795

